# Predictive modeling and ranking of physicochemicals in *Aegle marmelos* using topological indices and multi-criteria decision-making techniques

**DOI:** 10.3389/fchem.2026.1740913

**Published:** 2026-04-10

**Authors:** Aditya Narayan Bhandari, Jaganathan B

**Affiliations:** 1 School of Computer Science and Engineering, Vellore Institute of Technology, Chennai, Tamil Nadu, India; 2 Department of Mathematics, School of Advanced Sciences, Vellore Institute of Technology, Chennai, Tamil Nadu, India

**Keywords:** *Aegle marmelos* (bael), drug discovery, molecular weight, multi-criteria decision-making, multiple linear regression, phytochemicals, quantitative structure–property relationship, riboflavin

## Abstract

*Aegle marmelos* (Bael Patra) contains diverse bioactive phytochemicals with significant therapeutic potential. However, it is observed that systematic computational methods for their evaluation remain limited. In this study, an integrated framework has been devised which combines quantitative structure-property relationship(QSPR) modelling and multi-criteria decision-making (MCDM) techniques to analyse and rank 15 key phytochemicals. A set of six physicochemical properties, such as molecular weight (MW), topological polar surface area (TPSA), hydrogen bond donor (HBD) and acceptor (HBA) counts, melting point (MP), and rotatable bond count (RBC) were modelled using 16 adriatic topological indices through pairwise multiple linear regression. The statistically robust models with R^2^ ≥ 0.99 were retained. The frequency of the occurrence of descriptors in these models was used to derive objective weights, which were used in VIKOR,SAW and TOPSIS methods for ranking of compounds. The results demonstrate high predictive accuracy, with MW showing the strongest predictability and the inverse sum indeg (ISI) index emerging as the most influential descriptor. The three MCDM methods showed strong agreement, consistently identifying riboflavin, ellagic acid, and kaempferol as the top-ranked compounds. The unified approach, linking regression-based descriptor importance with decision making techniques, provides a robust and scalable approach for compound prioritization. The study possesses potential applications in virtual screening and early-stage drug discovery of plant derived molecules.

## 1 Introduction

*Aegle marmelos*, commonly referred to as bael, is a deciduous tree native to India and a member of the Rutaceae family (Monika et al., 2023). It typically grows to a height of 12 m–15 m and can withstand harsh dry climates (Baliga et al., 2011). Bael is considered a sacred plant in Hinduism and is widely used in traditional religious practices. In addition to its cultural and religious significance, bael is renowned for its numerous medicinal properties. The leaves and fruit pulp are rich in a wide array of bioactive compounds, including phenolics, pectins, carotenoids, alkaloids, coumarins, tannins, flavonoids, and terpenoids (Baliga et al., 2011).

The medicinal benefits of *A. marmelos* are largely attributed to its high concentration of phytochemicals. Key coumarins identified in the plant include aegeline, alloimperatorin, aegelenine, marmelin, O-methyl halfordinol, furocoumarins, psoralen, O-isopentenyl halfordinol, and marmelosin, as stated by Yadav and Rana (2025). Additional compounds present in the plant include tartaric acid, linoleic acid, phlobatannins, flavon-3-ols, leucoanthocyanins, anthocyanins, and flavonoid glycosides, as stated by Yadav and Rana (2025). The isolation of these bioactive constituents has enabled their use in the treatment of a variety of ailments. The chemical constituents of bael have been used in the treatment of a range of chronic conditions, such as diabetes, cancer, ulcers, inflammation, depression, stress, thyroid disorders, asthma, cardiovascular diseases, diarrhea, dysentery, and other related illnesses (Banerjee et al., 2024). Moreover, some studies have indicated that bael may contribute to the control of hyperlipidemia by aiding in the reduction of cholesterol levels (Mujeeb et al., 2025).

A nutritional analysis of the fruit reveals the following composition per 100 g of edible portion: 61.5 g moisture, 1.8 g protein, 0.39 g fat, 31.8 g carbohydrates, 1.7 g minerals, 55 mg carotene, 0.13 mg thiamine, 1.19 mg riboflavin, 1.1 mg niacin, and 8.0 mg vitamin C, as stated by Singh et al. (2019). It is used in Ayurveda and other traditional medicinal systems. The fruit contains various vitamins such as vitamin A, vitamins B1 and $B_{2}$ , and vitamin C. It is also rich in minerals such as potassium, phosphorus, iron, and calcium (Khanal et al., 2023).

In India and other countries, the development and integration of herbal and phytochemical-based therapies face significant regulatory challenges, including inconsistent quality control, limited safety and efficacy evaluations, and a lack of standardized pharmacovigilance systems (Ekor, 2014). These challenges underscore the need for alternative strategies such as computational and mathematical modeling methods to prioritize and evaluate the therapeutic potential of bael-based compounds. Moreover, methods such as high-throughput screening (HTS) are difficult to implement due to the high costs associated with maintaining high-quality screening libraries in pharmaceutical companies (Macarron et al., 2011). It has also been observed that early compound collections used for HTS, which were developed in the 1990s, often lacked consideration for the long-term sustainability of the compounds in drug discovery (Macarron et al., 2011). Given the limitations of traditional chemical testing, mathematical and computational models offer promising alternatives for evaluating the bioactivity of plant-derived compounds. Hence, it is observed that computational techniques such as quantitative structure–property relationship (QSPR) and quantitative structure–activity relationship (QSAR) analysis play an essential role in studying the bioactivity of various chemical compounds. Furthermore, certain novel techniques, as presented by Lionta et al. (2014), in which VS protocols were developed to increase inhibitor selectivity, also play an essential role in structure-based drug discovery.

In modern drug discovery, QSAR and QSPR modeling play vital roles in the prediction and analysis of molecular design. In the present day, chemo-informatics and ML-based QSAR play essential roles in drug discovery and development (Niazi and Mariam, 2023). Sathyanarayanan and Jaganathan (2023) demonstrated that the discrete Adriatic indices of a boron triangular sheet (BTS) can be effectively used to predict key physicochemical properties such as heat capacity, octanol–water partition coefficient, total surface area (TSA), and standard enthalpy of vaporization for sheets of arbitrary length and breadth. The versatility of QSAR modeling allows it to be integrated with machine learning concepts, which can help better facilitate the selection of appropriate strategies, develop existing methods, and provide platforms for studying existing methodologies (Keyvanpour and Shirzad, 2020). Similarly, the various chemical constituents of bael may be studied with QSPR and QSAR analyses. Joy Prisca and Jaganathan (2025) theoretically analyzed the physicochemical properties of internally functionalized dendrimers using topological descriptors, highlighting their potential for drug delivery in presenting an approach that may inform nanocarrier design for *A. marmelos* bioactives. In this study, we analyze various chemical constituents of *A. marmelos* such as auraptene, aegeline, kaempferol, ferulic acid, ellagic acid, umbelliferone, 1,8-cineole, acetoin, citronellal, eugenol, oxalic acid, tembamide, riboflavin, xanthotoxin, and ascorbic acid present in the Aegle marmelos plant that will help better determine their medicinal and pharmaceutical properties. Topological indices (TIs) and multi-criteria decision-making (MCDM) methods can be effectively utilized to achieve the aforementioned objective. Harsha Vardhan et al. (2023) performed regression analysis using indices, which demonstrated promising results. Enhanced discriminative power was observed when validation was performed for polycyclic aromatic hydrocarbons. Certain cost-effective approaches have contributed to the analogous prediction, optimization, and virtual screening of neem compounds, showing how insights may be gained into the properties of the constituent compounds and accelerating drug discovery and development (Anuradha and Jaganathan, 2025). Similar methods, as those described by Anuradha and Jaganathan (2023), analyze the physicochemical properties of benzophenone and curcumin-conjugated PAMAM dendrimers using TIs. It is further observed that anti-cancer drugs may be considered for further study on the basis of calculated TIs values (Shanmukha et al., 2020). Shirakol et al. (2019) demonstrated how a combination of distance-based indices, such as the Wiener index, terminal Wiener index, and hyper Wiener index, along with degree-distance-based indices, such as the Gutman index, Ashwini index, and SNM index, may be used for modeling the physical properties of compounds.

MCDM methods may be utilized along with QSAR and QSPR analyses to obtain better predictions in drug discovery. These approaches enable researchers to rank chemical compounds based on significant metrics such as toxicity and other physicochemical properties. A set of existing MCDM techniques includes VlseKriterijumska Optimizacija I Kompromisno Resenje (VIKOR), as introduced by Opricovic and Tzeng (2004), along with other techniques such as TOPSIS (technique for order preference by similarity to ideal solution), as introduced by Hwang and Yoon (1981). Other existing methods include the simple additive weight (SAW) ranking methods, as introduced by Churchman et al. (1957). Farooq et al. (2025) ranked a set of drug candidates using the technique for order preference by similarity to the ideal solution (TOPSIS) and two weighted aggregate sum product assessment (WASPAS) methods. Husin et al. (2024) used decision-making techniques such as COPRAS and PROMETHEE-II to rank medicines for kidney cancer therapies.

In this study, we aim to explore the correlation between the Adriatic TIs for 15 different phytochemicals present in the bael (*A. marmelos*) plant—viz., auraptene, aegeline, kaempferol, ferulic acid, ellagic acid, umbelliferone, 1,8-cineole, acetoin, citronellal, eugenol, oxalic acid, tembamide, riboflavin, xanthotoxin, and ascorbic acid—and a set of six physical properties, namely, melting point (MP), molecular weight (MW), topological polar surface area (TPSA), hydrogen bond donor (HBD) count, hydrogen bond acceptor (HBA) count, and rotatable bond count (RBC). *A. marmelos* is known to contain a diverse array of phytochemicals, including coumarins, flavonoids, terpenoids, alkaloids, phenolic acids, and vitamins, as reported in previous phytochemical studies (Choudhary et al., 2021; Narender et al., 2007). For QSPR modeling, these 15 compounds were selected because reliable and complete physicochemical data for them are readily available in curated databases such as PubChem and because they represent some of the most pharmacologically relevant bioactive constituents of the bael plant (Monika et al., 2023; Choudhary et al., 2021).

Furthermore, MCDM is used to rank the aforementioned compounds on the basis of their topological index values. Among the many topological descriptors available, the discrete Adriatic indices that comprise over 148 distinct bond-additive measures represent one of the most comprehensive and versatile families of degree-based molecular descriptors. These indices effectively capture variations in branching, local connectivity, and atomic environments within molecular graphs, making them highly suitable for QSPR modeling and compound prioritization tasks (Vukičević and Gašperov, 2010). For several physicochemical properties, these indices have demonstrated excellent predictive performance in QSPR modeling, frequently surpassing classical descriptors such as the Wiener or Zagreb indices (Vukicevic, 2011). Their established success in drug-likeness evaluation and modeling of biomolecular and nanostructured systems makes discrete Adriatic descriptors a robust and reliable framework for analyzing the selected phytochemicals in this study.

To the best of our knowledge, this is the first study to utilize a unified framework that directly links QSPR analysis to MCDM for the prioritization of *A. marmelos* phytochemicals. The aim of this work is twofold: first, to establish statistically robust QSPR models by identifying the optimal pairwise combinations of Adriatic TIs that best predict the six selected physicochemical properties (specifically targeting models with $R^{2}\geq 0.99$ ); second, to utilize the predictive strength of these optimal TIs as objective data-driven weights for the VIKOR, SAW, and TOPSIS MCDM techniques to generate a highly reliable and consensus-based ranking of the 15 *A. marmelos* phytochemicals based on their therapeutic potential.

In summary, in this study, we demonstrate that discrete Adriatic indices offer a refined and structurally sensitive approach to QSPR modeling, effectively capturing branching and molecular complexity. Their integration with objective regression-derived weights in MCDM techniques provides a robust framework for compound prioritization.

## 2 Phytochemicals in *Aegle marmelos*

*A. marmelos*, colloquially referred to as bael, is constituted of a vast array of phytochemicals. Bael is rich in a series of physicochemicals such as alkaloids, flavonoids, coumarins, tannins, terpenoids, phenolics, and saponins. Rahman et al. (2024) conducted a series of experiments to accurately determine the composition of phenols, flavonoids, tannins, reducing sugar content, and non-reducing sugar content in bael. This study focuses on analyzing 15 chemical constituents of *A. marmelos*, including ellagic acid, ferulic acid, kaempferol, aegeline, and auraptene (Choudhary et al., 2021; Narender et al., 2007; Monika et al., 2023), along with umbelliferone, 1,8-cineole, acetoin, citronellal, eugenol, oxalic acid, tembamide, riboflavin, xanthotoxin, and ascorbic acid (Choudhary et al., 2021).

Although a large number of phytochemicals have been reported in *A. marmelos*, as shown above, this study restricts itself to 15 compounds. These molecules were selected because their structures and physicochemical properties are well-documented in curated databases such as PubChem, enabling reliable QSPR analysis. Additionally, these 15 compounds represent the most pharmacologically relevant bioactive classes of bael—including flavonoids, coumarins, phenolic acids, alkaloids, terpenoids, and vitamins—which have been consistently associated with antioxidant, antimicrobial, anti-inflammatory, antidiabetic, and hepatoprotective activities (Choudhary et al., 2021; Monika et al., 2023; Narender et al., 2007). Thus, the present dataset reflects both the data availability and biological relevance, thus ensuring a representative and analytically consistent selection for predictive modeling.

**FIGURE 1:**
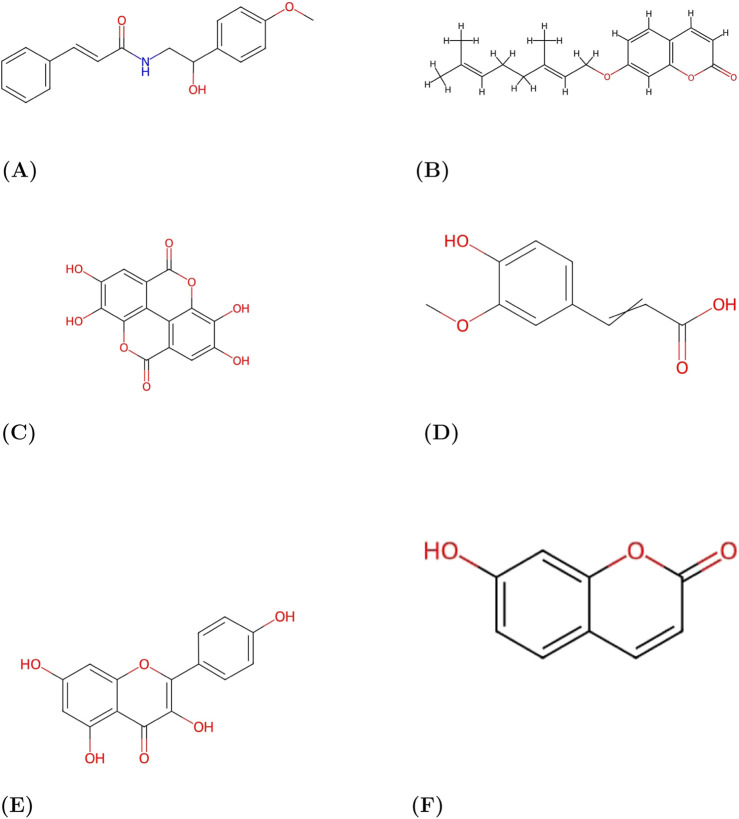
Chemical structures part 1. Representative chemical structures of phytochemicals in *Aegle marmelos*. **(A)** Aegeline. **(B)** Auraptene. **(C)** Ellagic acid. **(D)** Ferulic acid. **(E)** Kaempferol. **(F)** Umbelliferone.

**FIGURE 2:**
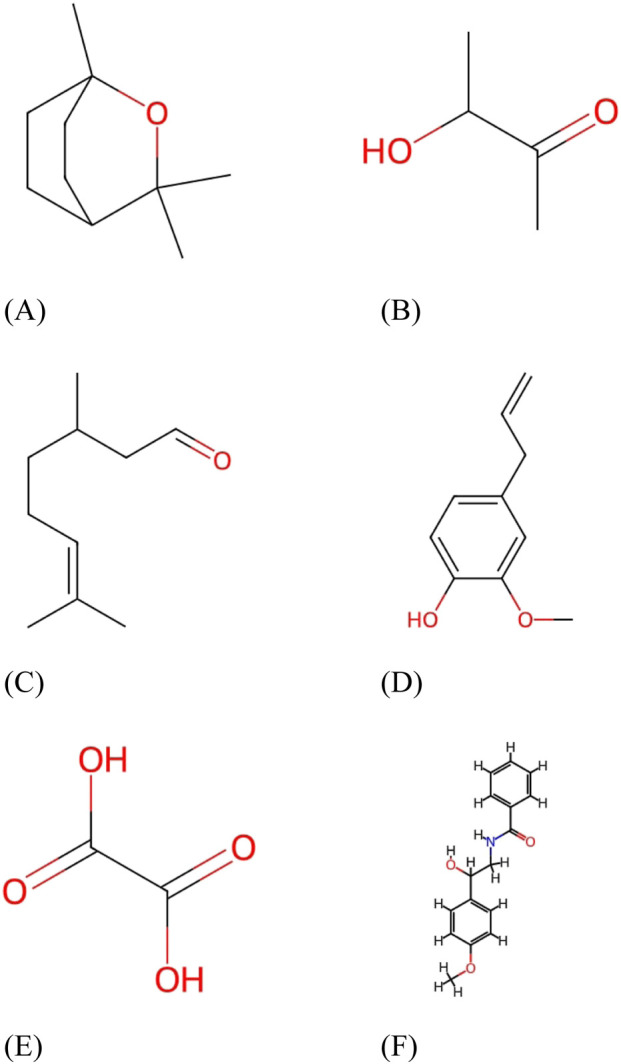
Chemical structures part 2. Representative chemical structures of phytochemicals in *Aegle marmelos*. **(A)** 1,8-Cineole. **(B)** Acetoin. **(C)** Citronellal. **(D)** Eugenol. **(E)** Oxalic acid. **(F)** Tembamide.

**FIGURE 3:**
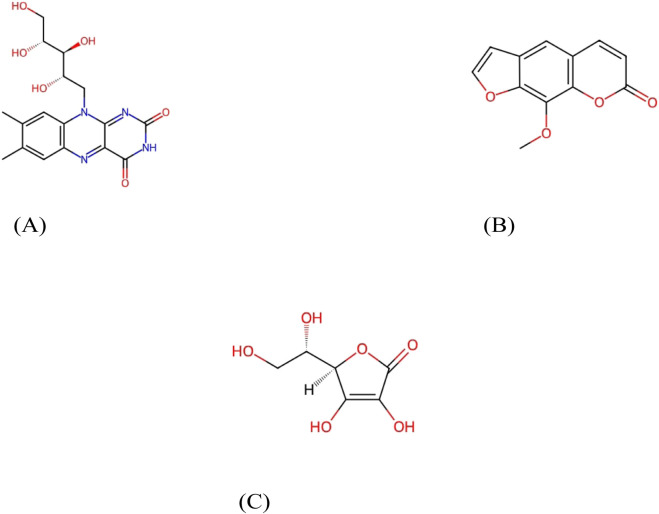
Chemical structures part 3. Representative chemical structures of phytochemicals in *Aegle marmelos*. **(A)** Riboflavin. **(B)** Xanthotoxin. **(C)** Ascorbic acid.

The chemical structures illustrated in Figures 1–3 correspond to the phytochemical constituents identified in *A. marmelos*. Figure 1A presents aegeline (ChemSpider, 2025a), while Figure 1B shows auraptene (ChemSpider, 2025b). Ellagic acid (ChemSpider, 2025c) and ferulic acid (ChemSpider, 2025d) are depicted in Figures 1C,D, respectively, followed by kaempferol (ChemSpider, 2025e) and umbelliferone (Royal Society of Chemistry, 2025h) in Figures 1E,F, respectively.

Similarly, Figure 2A illustrates 1,8-cineole (Royal Society of Chemistry, 2025e), Figure 2B presents acetoin (Royal Society of Chemistry, 2025d), Figure 2C shows citronellal (Royal Society of Chemistry, 2025i), and Figure 2D depicts eugenol (Royal Society of Chemistry, 2025b). Oxalic acid (Royal Society of Chemistry, 2025j) and tembamide (Royal Society of Chemistry, 2025c) are displayed in Figures 2E,F, respectively.

Furthermore, riboflavin (Royal Society of Chemistry, 2025g), xanthotoxin (Royal Society of Chemistry, 2025f), and ascorbic acid (Royal Society of Chemistry, 2025a) are illustrated in Figures 3A–C, respectively.

## 3 Valency-based indices and drug-likeness prediction

In QSPR and QSAR, various physicochemical properties are essential as they help govern a molecule’s ADMET profile. ADMET stands for absorption, distribution, metabolism, excretion, and toxicity. Some essential physicochemical properties that are essential for drug prediction are molecular weight, log(P) (partition coefficient), topological polar surface area, hydrogen bond donors, hydrogen bond acceptors, rotatable bond count, boiling point, melting point, water solubility, refractive index, density, pKa, volume, and surface area, among others. However, it is observed that the ADMET profile of *A. marmelos* is not well known and has not been extensively studied.

This study focuses on the role of melting point, molecular weight, topological polar surface area, hydrogen bond donor count, hydrogen bond acceptor count, and rotatable bond count in the efficacy of pharmaceutical compounds and the predictive analysis of drug behavior. The melting point is one of the earliest and most reliable physical properties measured during drug development. The high significance of the melting point of drugs lies in the fact that its determination does not require complex procedures or pKa measurement, providing a quick and accessible means to guide early-stage compound selection. Chu and Yalkowsky (2009) related the melting point to the intrinsic solubility and the partition coefficient of a compound via the general solubility equation. This relationship allowed the melting point to serve as a proxy for understanding solubility–permeability dynamics in passive absorption models. It is observed that a lower melting point is associated with higher absorption, while a higher melting point tends to correlate with lower absorption, particularly as the drug dose increases (Chu and Yalkowsky, 2009). Molecular weight also plays an essential role in studying drug behavior. Toews and Bates (2023) studied the effect of drug molecular weight on the release kinetics from HEMA and HPMA hydrogels. It was found that molecular weight governs drug–hydrogel compatibility, balancing release efficiency, stability, and optical/mechanical performance. Additionally, MW is a key structural descriptor that affects the solubility, permeability, binding affinity, and drug-likeness. Molecular weight directly affects absorption, distribution, metabolism, and excretion (ADME) properties, as demonstrated by Shino and Kaneko (2025). The polar surface area (PSA) refers to the surface associated with heteroatoms and polar hydrogen atoms in a molecule, excluding nonpolar elements such as carbon and halogens. Prasanna and Doerksen (2009) demonstrated how the use of TPSA provides a practical estimate of a molecule’s polar surface area using functional group contributions from a large structural database, eliminating the need for 3D structural calculations or conformational analysis. This study also emphasizes the role of hydrogen bond donor and acceptor counts, which are key molecular descriptors commonly used to estimate the oral bioavailability of small-molecule drugs. These counts are integral to widely accepted drug-likeness rules, such as Lipinski’s rule of five (Ro5) and the Veber criteria, due to their significant influence on passive membrane permeability, which directly influences drug absorption and distribution (Coimbra et al., 2021). Balali et al. (2021) examined how variations in HBD structures influence the distribution of thiophene between the choline chloride-based deep eutectic solvent and hydrocarbon phases in ternary systems. From a QSPR perspective, the HBD count serves as a predictive variable that correlates with the distribution coefficient of thiophene. In this case, varying the HBD structure—and thus the number and position of the hydrogen bond donor groups—enables the model to capture how structural changes in DES components influence extraction efficiency (Balali et al., 2021). Hydrogen bonding is also immensely important in enzymatic catalysis. The hydrogen bond acceptor count serves as a fundamental molecular descriptor that reflects how a compound interacts with its environment—particularly through solvation and hydrogen bonding. According to Mercader et al. (2010), the enhanced replacement method identified HBA count as one of the top-four descriptors in the optimal model, emphasizing its quantitative contribution to the property being modeled. The HBA count helps predict absorption, distribution, and permeability as hydrogen bonding influences a compound’s ability to cross biological membranes. The number of rotatable bonds refers to the number of non-ring, non-terminal single bonds, typically between non-hydrogen atoms (often sp^3^–sp^3^ C–C bonds), that allow free rotation. It quantifies the flexibility of a molecule in terms of conformational freedom. NRot remains a core descriptor in classical QSPR and QSAR models, particularly within the Ro5-compliant drug space (Caron et al., 2020).

The physicochemical properties of the chosen chemical compounds are provided in Table 1.

**TABLE 1:** Physicochemical properties of selected *Aegle marmelos* compounds.

Compound	MP (°C)	MW (g/mol)	TPSA (Å^2^)	HBD	HBA	RBC
Aegeline	176	297.3	58.6	2	3	6
Auraptene	68	298.4	35.5	0	3	6
Ellagic acid	> 360	302.19	134	4	8	0
Ferulic acid	168	194.18	66.8	2	4	3
Kaempferol	277	286.24	107	4	6	1
Umbelliferone	230	162.14	46.5	1	3	0
1,8-Cineole	1.5	154.25	9.2	0	1	0
Acetoin	15	88.11	37.3	1	2	1
Citronellal	147	154.25	17.1	0	1	5
Eugenol	−9.22	164.20	29.5	1	2	3
Oxalic acid	188.89	90.03	74.6	2	4	1
Tembamide	156	271.31	58.6	2	3	5
Riboflavin	280	376.4	155	5	7	5
Xanthotoxin	147.78	216.19	48.7	0	4	1
Ascorbic acid	190	176.12	107	4	6	2

The melting point and other physicochemical properties of the selected compounds were compiled from authoritative databases. Specifically, the melting point of aegeline is sourced from the Human Metabolome Database (2025a), and the remaining properties are sourced from PubChem (2025b). Data for auraptene are obtained from the Human Metabolome Database (HMDB) (2022) and PubChem (2025d). For ellagic acid, the melting point is cited from the National Toxicology Program (1992), while other properties are taken from the National Center for Biotechnology Information (2025b). Ferulic acid values are sourced from the Human Metabolome Database (2025b) and the National Center for Biotechnology Information (2025a). All parameters for kaempferol, umbelliferone, 1,8-cineole, acetoin, citronellal, eugenol, oxalic acid, tembamide, riboflavin, xanthotoxin, and ascorbic acid are retrieved from PubChem (2025h), PubChem, N (2025), PubChem (2025f), PubChem (2025a), PubChem (2025e), PubChem (2025g), PubChem (2025j), PubChem (2025L), PubChem (2025k), PubChem (2025i), and PubChem (2025c), respectively.

The discrete Adriatic topological indices form the most widely applied subclass of the Adriatic framework. In total, the Adriatic index family comprises more than 148 discrete descriptors. From this extensive set, 16 representative indices—Randic-type lodeg index (RLI), sum lordeg index (SLI), inverse sum lordeg index (ISLI), misbalance lodeg index (MLI), misbalance losdeg index (MLSI), misbalance indeg index (MII), misbalance irdeg index (MIRI), misbalance rodeg index (MRI), misbalance deg index (MDI), misbalance hadeg index (MHI), min–max rodeg index (MMRI), max–min rodeg index (MMRDI), max–min deg index (MMDI), max–min Sdeg index (MMSDI), symmetric division deg index (SDDI), and inverse sum indeg (ISI) index—as presented by Anuradha. et al. (2024), were selected for this study. For future works, some additional descriptors, as mentioned by Anuradha and Jaganathan (2025), may be utilized to study the physicochemical properties of chemical compounds. Discrete Adriatic descriptors are capable of sensitively capturing branching and local bonding environments (Vukičević and Gašperov, 2010). These indices have demonstrated strong QSPR performance, with several topological indices achieving high correlation for a range of physicochemical properties such as enthalpy of vaporization, logP, density, and total surface area. These discrete Adriatic indices often outperform classical indices such as the Wiener or Zagreb descriptors (Vukicevic, 2011). Their simplicity in computation and proven success in modeling drug delivery dendrimers and nanostructured systems further justify their selection for phytochemical analysis (Anuradha and Jaganathan, 2023). The TIs are calculated using various analytical methods derived from graph theory, including edge partitioning, vertex partitioning, and strategies based on eccentricity and the vertex degree. A category of degree-based indices, known as discrete Adriatic indices, as described by Anuradha. et al. (2024), is used to analyze the given chemical structures. Throughout this study, Ω represents a chemical graph, E(Ω) denotes the set of all edges $\partial ,\partial_{1},\partial_{2},\partial_{3},\ldots$ , and V(Ω) denotes the set of all vertices $\alpha ,\alpha_{1},\alpha_{2},\alpha_{3},\ldots$ . Two neighboring vertices are connected by a link between them. An edge denoted by ∂ = $\alpha \alpha_{1}$ represents an edge in E (Ω) . The number of edges that are incident to any vertex α is the degree of vertex and is denoted by $d_{\alpha }$ . The topological indices that have been used are based on the edge-partition technique.

### 3.1 Discrete Adriatic indices

This study evaluates a set of 16 bond additive descriptor indices based on physicochemical properties, as described by Anuradha et al. (2024). The index formations, along with their respective definitions, are presented in the following points. In this study, we analyze a collection of 16 degree-based topological indices belonging to the class of *discrete Adriatic indices* based on their reported effectiveness in modeling physicochemical and thermodynamic properties of molecular structures (Anuradha et al., 2024). These indices are utilized as bond-additive descriptors, meaning that they are computed as sums over the edges of a molecular graph Ω=(V,E) , where the vertices represent atoms, the edges represent chemical bonds, and $d_{\alpha }$ denotes the degree of vertex α .

The Adriatic indices were originally introduced to provide a unified and systematically derivable framework for constructing topological descriptors that capture the local and global connectivity patterns beyond classical distance-based indices. Formally, an Adriatic index can be expressed in the general closed form

$$TI\left(\Omega \right)=\underset{uv\in E\left(\Omega \right)}{\sum }\gamma \left(\phi \left(d_{u}\right),\phi \left(d_{v}\right)\right),$$

where ϕ(d) is a degree-transformation function and γ(x,y) is a symmetric binary operator. Different combinations of ϕ and γ generate distinct families of bond-additive indices. All descriptors considered in this study arise as special cases of this general formulation, thus ensuring mathematical consistency, reproducibility, and structural interpretability.

From a structural perspective, Adriatic indices are designed to encode variations in local bonding environments, branching irregularities, and degree heterogeneity within molecular graphs. Transformations such as logarithmic, reciprocal, square-root, or exponential decay emphasize nonlinear connectivity effects, while binary operators such as misbalance, inverse sum, and extremal ratios quantify the asymmetry or contrast between adjacent atomic environments. These properties are particularly relevant in QSPR and QSAR modeling, where subtle topological variations strongly influence thermodynamic quantities such as heat capacity, enthalpy of vaporization, density, and surface-related properties.

Recent advances in molecular informatics have increasingly combined graph-theoretic descriptors with machine-learning-based QSPR frameworks to enhance predictive accuracy and applicability in medicinal chemistry. For example, a recent hybrid computational framework for antidepressant drug design integrates eccentricity-based topological indices with linear regression, random forest, and XGBoost models, demonstrating improved prediction of physicochemical properties and drug-relevant features. Similarly, machine-learning-assisted studies utilizing artificial neural networks and random forest models have shown that topological indices are highly informative descriptors for predicting properties such as the enthalpy of vaporization, molar refractivity, density, and polar surface area, particularly when nonlinear structure–property relationships are present (Ahmed et al., 2025a; Ahmed et al., 2025b).

These studies highlight the growing trend toward hybrid descriptor–machine learning frameworks in drug discovery. In contrast, the present work emphasizes a statistically rigorous and interpretable QSPR strategy based on systematically derived Adriatic topological indices combined with regression analysis and MCDM-based prioritization. This complementary approach retains chemical interpretability while providing robust predictive performance, making it suitable for data-driven compound screening where transparency and decision rationality are essential.

Based on their underlying functional construction, the Adriatic indices used in this work may be broadly classified as follows:

- Log-degree-based indices (e.g., RLI, SLI, and ISLI), which employ logarithmic transformations of vertex degrees to enhance sensitivity to nonlinear connectivity effects and local structural variations.
- Misbalance descriptors (e.g., MLI, MLSI, MII, MIRI, MRI, MDI, and MHI), which compute absolute differences between degree-based quantities across adjacent vertices, thereby quantifying the topological asymmetry and branching irregularity.
- Ratio-based and extremal indices (e.g., MMRI, MMRDI, MMDI, MMSDI, and SDDI), which emphasize minimum–maximum or maximum–minimum degree contrasts, reflecting extremal connectivity behavior within molecular substructures.
- Inverse-sum and decay-type indices (e.g., ISI), which attenuate the contribution of high-degree vertices and mimic the physical decay processes associated with diffusion, accessibility, or reactive zones.

Due to their systematic derivation and enhanced structural resolution, Adriatic indices have been shown to outperform several classical descriptors such as the Wiener and Zagreb indices in predicting physicochemical properties within QSPR frameworks (Vukičević and Gašperov, 2010; Vukicevic, 2011). In the following, we present the explicit definitions of each index utilized in this study, followed by their computed values summarized in Table 2. All indices except RLI arise as special cases of the Adriatic index framework (Vukičević and Gašperov, 2010) and are, therefore, not cited individually.

- Randic-type lodeg index (RLI) (Randic, 1975)—a good interpreter of heat capacity.
$$RLI\left(\Omega \right)=\underset{\partial \in E\left(\Omega \right)}{\sum }\ln\left(d_{\alpha }\right)\ln\left(d_{\alpha_{1}}\right).$$
- Sum lordeg index (SLI)—effectively interprets the Log-P value.
$$SLI\left(\Omega \right)=\underset{\partial \in E\left(\Omega \right)}{\sum }\left[\sqrt{\ln\left(d_{\alpha }\right)}+\sqrt{\ln\left(d_{\alpha_{1}}\right)}\right].$$
- Inverse sum lordeg index (ISLI)—useful for interpreting the TSA.
$$ISLI\left(\Omega \right)=\underset{\partial \in E\left(\Omega \right)}{\sum }\frac{d_{\alpha }d_{\alpha_{1}}}{d_{\alpha }+d_{\alpha_{1}}}.$$
- Misbalance lodeg index (MLI)—correlates well with the enthalpy of vaporization.
$$MLI\left(\Omega \right)=\underset{\partial \in E\left(\Omega \right)}{\sum }\left|\ln\left(d_{\alpha }\right)-\ln\left(d_{\alpha_{1}}\right)\right|.$$
- Misbalance losdeg index (MLSI)—another index linked to the enthalpy of vaporization.
$$MLSI\left(\Omega \right)=\underset{\partial \in E\left(\Omega \right)}{\sum }\left|\ln^{2}\left(d_{\alpha }\right)-\ln^{2}\left(d_{\alpha_{1}}\right)\right|.$$
- Misbalance indeg index (MII)—found to interpret biological activity.
$$MII\left(\Omega \right)=\underset{\partial \in E\left(\Omega \right)}{\sum }\left|\frac{1}{d_{\alpha }}-\frac{1}{d_{\alpha_{1}}}\right|.$$
- Misbalance irdeg index (MIRI)—associated with the enthalpy of vaporization.
$$MIRI\left(\Omega \right)=\underset{\partial \in E\left(\Omega \right)}{\sum }\left|\frac{1}{\sqrt{d_{\alpha }}}-\frac{1}{\sqrt{d_{\alpha_{1}}}}\right|.$$
- Misbalance rodeg index (MRI)—an index for the standard enthalpy of vaporization.
$$MRI\left(\Omega \right)=\underset{\partial \in E\left(\Omega \right)}{\sum }\left|\sqrt{d_{\alpha }}-\sqrt{d_{\alpha_{1}}}\right|.$$
- Misbalance deg index (MDI)—also reflects the standard enthalpy of vaporization.
$$MDI\left(\Omega \right)=\underset{\partial \in E\left(\Omega \right)}{\sum }\left|d_{\alpha }-d_{\alpha_{1}}\right|.$$
- Misbalance hadeg index (MHI)—represents the standard enthalpy of vaporization.
$$MHI\left(\Omega \right)=\underset{\partial \in E\left(\Omega \right)}{\sum }\left|{\left(\frac{1}{2}\right)}^{d_{\alpha }}-{\left(\frac{1}{2}\right)}^{d_{\alpha_{1}}}\right|.$$
- Min–max rodeg index (MMRI)—serves as a good indicator for enthalpy.
$$MMRI\left(\Omega \right)=\underset{\partial \in E\left(\Omega \right)}{\sum }\sqrt{\frac{\min\left(d_{\alpha },d_{\alpha_{1}}\right)}{\max\left(d_{\alpha },d_{\alpha_{1}}\right)}}.$$
- Max–min rodeg index (MMRDI)—interprets density effectively.
$$MMRDI\left(\Omega \right)=\underset{\partial \in E\left(\Omega \right)}{\sum }\sqrt{\frac{\max\left(d_{\alpha },d_{\alpha_{1}}\right)}{\min\left(d_{\alpha },d_{\alpha_{1}}\right)}}.$$
- Max–min deg index (MMDI)—related to the relative retention time.
$$MMDI\left(\Omega \right)=\underset{\partial \in E\left(\Omega \right)}{\sum }\frac{\max\left(d_{\alpha },d_{\alpha_{1}}\right)}{\min\left(d_{\alpha },d_{\alpha_{1}}\right)}.$$
- Max–min Sdeg index (MMSDI)—also interprets the relative retention time.
$$MMSDI\left(\Omega \right)=\underset{\partial \in E\left(\Omega \right)}{\sum }{\left(\frac{\max\left(d_{\alpha },d_{\alpha_{1}}\right)}{\min\left(d_{\alpha },d_{\alpha_{1}}\right)}\right)}^{2}.$$
- Symmetric division deg index (SDDI)—a useful indicator for TSA.
$$SDDI\left(\Omega \right)=\underset{\partial \in E\left(\Omega \right)}{\sum }\left(\frac{\min\left(d_{\alpha },d_{\alpha_{1}}\right)}{\max\left(d_{\alpha },d_{\alpha_{1}}\right)}+\frac{\max\left(d_{\alpha },d_{\alpha_{1}}\right)}{\min\left(d_{\alpha },d_{\alpha_{1}}\right)}\right).$$
- Inverse sum indeg index (ISI)—another index that correlates well with heat capacity.

$$ISI\left(\Omega \right)=\underset{\partial \in E\left(\Omega \right)}{\sum }\frac{1}{\sqrt{\ln\left(d_{\alpha }\right)}+\sqrt{\ln\left(d_{\alpha_{1}}\right)}}.$$

**TABLE 2:** Calculation of the values of topological indices for compounds in *Aegle marmelos*.

TI	Aegeline	Auraptene	Ellagic acid	Ferulic acid	Kaempferol	Umbelliferone	1,8-Cineole	Acetoin	Citronellal	Eugenol	Oxalic acid	Tembamide	Riboflavin	Xanthotoxin	Ascorbic acid
RLI	13.4271	13.2277	19.3684	7.4984	15.8176	8.2599	7.8505	1.2069	3.7259	6.7369	1.2069	12.6306	19.5551	13.1227	7.1123
SLI	39.0337	38.8480	44.3937	22.5684	39.9557	22.5684	20.8893	6.2889	14.6144	19.4240	6.2889	35.7035	49.3891	32.1875	19.0524
ISLI	24.8667	24.9000	30.6000	14.8167	26.7500	14.6000	13.8476	4.5000	9.5167	12.7833	4.5000	22.9667	33.5167	21.2167	13.2667
MLI	7.3505	9.2600	9.8354	6.8272	9.5477	5.4409	8.0301	4.3944	5.2054	4.9177	4.3944	6.5396	13.6545	5.4409	6.3040
MLSI	13.8470	15.8621	26.6790	10.9070	21.0303	8.0827	8.0022	4.8278	6.7369	7.9311	6.0347	12.0080	26.3375	13.2265	8.4359
MII	3.6667	4.6667	5.3333	3.6667	5.0000	2.6667	3.6667	2.6667	3.0000	2.6667	2.6667	3.3333	7.3333	2.6667	3.6667
MIR	2.6665	3.2477	3.6574	2.4691	3.4108	1.8834	2.6663	1.6906	1.9601	1.7867	1.6906	2.3060	4.9383	1.8834	2.3728
MRI	5.3745	6.7423	6.9350	4.8352	6.8886	4.0068	6.2468	2.9928	3.5639	3.4676	2.9928	4.7389	9.6705	4.0068	4.2959
MDI	16.000	20.000	20.000	14.000	20.000	12.000	20.000	8.0000	10.000	8.0000	14.000	28.000	12.000	12.000	12.000
MHI	2.3750	3.0000	3.2500	2.2500	3.1250	1.7500	2.3750	1.5000	1.7500	1.6250	1.5000	2.1250	4.5000	1.7500	2.1250
MMDI	19.8433	19.1074	20.9961	11.1546	19.0517	10.6867	8.8774	3.3094	7.8886	5.8906	3.3094	18.2103	23.3361	15.6329	9.4660
MMDII	27.3506	28.6251	31.1903	18.1836	28.9077	16.2621	17.2610	7.9282	13.2846	14.9089	7.9282	24.9010	37.4603	21.1690	16.0167
MMDI	33.500	37.000	41.000	24.500	38.000	21.000	26.3333	13.000	18.500	19.000	13.000	28.000	50.500	25.500	22.500
MSSD	56.750	70.000	88.300	49.750	85.500	39.000	72.2778	37.000	40.750	33.500	37.000	51.250	104.250	40.250	50.750
SDI	51.000	53.3333	59.3333	33.6667	54.3333	30.000	48.1667	15.3333	26.000	27.3333	15.3333	46.6667	70.000	30.3333	30.8333
ISI	14.2397	14.2773	16.2355	9.4635	14.1508	7.8400	7.2519	4.2933	7.4601	8.2248	4.2933	13.0627	18.6081	10.6604	8.5207

The calculated values for TIs are presented as follows in Table 2.

**FIGURE 4:**
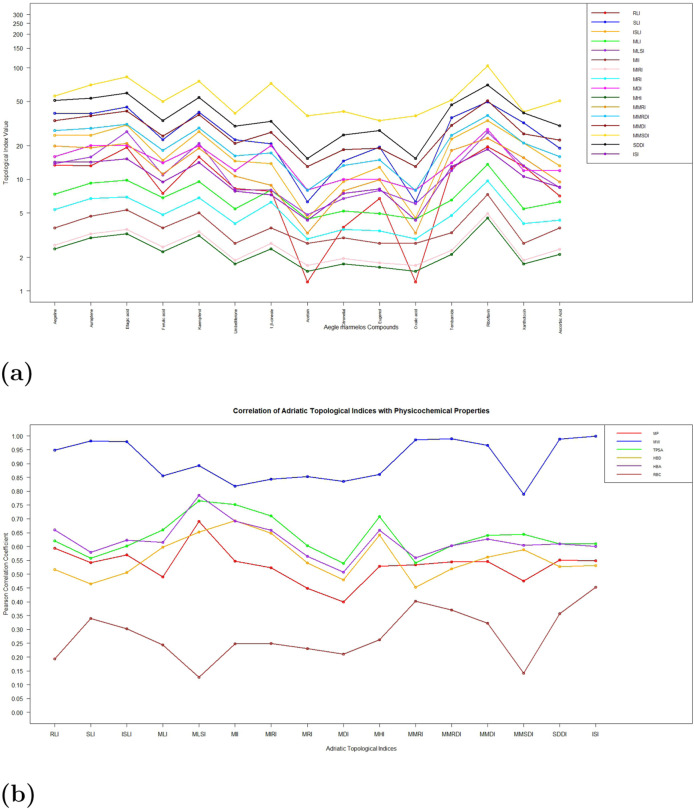
Graphical representation of the TIs and their correlation with the physicochemical properties of the *Aegle marmelos* compounds. **(a)** TIs and properties. **(b)** Correlation coefficients. **(a)** TIs and properties of *Aegle marmelos* compounds (graphical representation). **(b)** Correlation coefficient between the properties and TIs of *Aegle marmelos.*

For any QSPR model, the correlation coefficients serve as a crucial metric for determining the efficacy of the specified model. The correlation coefficients of the topological indices with respect to the corresponding physicochemical properties have been plotted in the following graphs, as presented in Figure 4. The values for the respective correlation coefficients have been presented in Table 3. Figure 4a presents a comparative graphical representation of the computed topological indices and physicochemical properties of the *A. marmelos* compounds, illustrating their variation across the dataset. Figure 4b shows the corresponding correlation coefficients between each topological index and the selected physicochemical properties. Stronger correlations are observed for size- and connectivity-dependent properties such as molecular weight and TPSA, while weaker correlations are noted for properties influenced by conformational flexibility, such as rotatable bond count. The numerical correlation values are summarized in Table 3.

**TABLE 3:** Correlation coefficients between the topological indices and physicochemical properties of *Aegle marmelos* compounds.

Topological index	MP	MW	TPSA	HBD	HBA	RBC
RLI	0.5942	0.9479	0.6207	0.5171	0.6596	0.1935
SLI	0.5413	0.9818	0.5575	0.4649	0.5796	0.3395
ISLI	0.5702	0.9788	0.6011	0.5063	0.6232	0.3023
MLI	0.4899	0.8550	0.6604	0.5969	0.6155	0.2435
MLSI	0.6912	0.8926	0.7648	0.6520	0.7848	0.1271
MII	0.5469	0.8186	0.7524	0.6927	0.6924	0.2474
MIRI	0.5229	0.8433	0.7109	0.6485	0.6585	0.2489
MRI	0.4484	0.8525	0.6026	0.5399	0.5648	0.2305
MDI	0.3996	0.8356	0.5392	0.4793	0.5078	0.2102
MHI	0.5289	0.8602	0.7075	0.6417	0.6583	0.2620
MMRI	0.5334	0.9863	0.5405	0.4526	0.5587	0.4025
MMRDI	0.5449	0.9903	0.6030	0.5188	0.6026	0.3697
MMDI	0.5452	0.9661	0.6401	0.5611	0.6267	0.3224
MMSDI	0.4746	0.7886	0.6437	0.5889	0.6041	0.1416
SDDI	0.5505	0.9879	0.6102	0.5265	0.6100	0.3571
ISI	0.5478	0.9988	0.6096	0.5314	0.6001	0.4524

As illustrated in Figure 4a, the graphical representation highlights the variation in TIs and physicochemical properties of the *A. marmelos* compounds, while Figure 4b presents the corresponding correlation coefficients between these parameters.

## 4 Regression model for the physicochemicals of *Aegle marmelos*

This section explores the quantitative relationships between selected topological indices and the six key physicochemical properties of *A. marmelos* compounds. Simple linear regression, quadratic regression, and multiple linear regression (MLR) techniques were utilized to identify the most effective predictive models. The models were used with the aim of assessing and comparing the ability of different topological descriptors to predict molecular properties such as molecular weight, topological polar surface area, and hydrogen bonding characteristics. To ensure statistical robustness, each MLR model was evaluated using standard significance criteria. Models with $R^{2}\geq 0.99$ and *p*-values <0.05 were considered statistically significant. Additional diagnostic checks for multicollinearity and residual behavior were performed during analysis to validate model assumptions. Among the 720 pairwise models developed from topological indices and physicochemical properties in the multiple linear regression model, only those meeting these criteria were selected and are presented in Table 6 and Equations 1–17. This allowed for the identification of the most predictive and reliable descriptor combinations for further analysis.

### 4.1 Considered modeling approaches

Three regression models were considered in this study, namely, (i) simple linear regression, (ii) quadratic regression, and (iii) multiple linear regression. Each physicochemical property was regressed individually against each topological index, followed by combined modeling with index pairs. The goal was to determine whether linear or non-linear patterns existed between the descriptors and molecular behavior, Regression performance was primarily evaluated using the coefficient of determination $\left(R^{2}\right)$ , and *p*-values were used to verify the statistical significance.

### 4.2 Simple linear regression analysis

Simple linear regression models were constructed to evaluate the individual influence of each TI on the physicochemical properties of the *A. marmelos* compounds. Each physicochemical property, namely, MW, MP, TPSA, HBDs, HBAs, and RBC, was regressed independently against each of the 16 TIs.

The form of regression equation used is stated as in Equation 1.

Y=A+B⋅TI, (1)

where Y represents a physicochemical property, TI denotes a single TI, A is the intercept, and B is the regression coefficient.

The statistical outputs from each model—including the coefficient of determination $\left(R^{2}\right)$ , *p*-value, intercept A , and slope B —were recorded for all combinations. A total of 96 simple linear regression models were computed. These results were ranked based on $R^{2}$ , and a selected subset of models with high predictive performance is summarized in Table 4. The complete set of linear regression results is provided in Supplementary Table S1 (linear_regression_all_models_sorted.csv).

**TABLE 4:** Simple linear regression model with $R^{2}\geq 0.99$ .

Dependent	Independent	$R^{2}$	*p*-value	A	B
MW	ISI	0.9975	$2.46\times 10^{-18}$	3.5920	20.1675

Although a few individual TI–property relationships yielded high $R^{2}$ values (e.g., MW–ISI and TPSA–MHI), the majority of the simple linear regression models demonstrated moderate-to-low predictive strength. This outcome indicates that no single topological index alone can fully explain the variation in physicochemical properties.

To address this limitation, MLR was subsequently applied using pairwise combinations of indices. The MLR approach yielded a substantially greater number of highly predictive models $\left(R^{2}>0.99\right)$ , highlighting the synergistic relationships between multiple indices and supporting the selection of MLR as the primary modeling framework for this study.

### 4.3 Quadratic regression analysis

Quadratic regression models were evaluated to explore potential non-linear relationships between the topological indices and the physicochemical properties of the *A. marmelos* compounds. In this model, each topological index was regressed against a physicochemical property using both the index and its squared term as the predictors.

The quadratic regression equation adopted is provided as follows in Equation 2.

$$PC=A+B\cdot TI+C\cdot TI^{2},$$ (2)

where PC denotes the physicochemical property, TI is the topological index, and A,B,C are the regression coefficients.

A total of 96 quadratic regression models were generated (one for each index–property combination). Among them, only one model achieved a coefficient of determination $\left(R^{2}\right)$ exceeding 0.99, which is presented in Table 5. This model demonstrates a strong predictive relationship between molecular weight and the ISI index in a non-linear form.

**TABLE 5:** Top quadratic regression models with $R^{2}\geq 0.99$ .

Dependent	Independent	$R^{2}$	*p*-value	A	B	C
MW	ISI	0.9976	$1.69\times 10^{-16}$	−1.867	21.327	−0.0529

While quadratic models improved the fit over simple linear regressions in certain cases, the limited number of models exceeding the $R^{2}\geq 0.99$ threshold indicates that single-index non-linear modeling remains insufficient for capturing the complexity of physicochemical behavior across the dataset. The complete list of quadratic regression models and their statistics is provided in the Supplementary Material (see quadratic_regression_all_models_sorted.csv).

### 4.4 Multiple linear regression analysis

MLR models were developed to comprehensively evaluate the combined effect of topological indices on the physicochemical properties. Unlike simple and quadratic regressions, which consider a single index per model, MLR incorporates two indices simultaneously, offering a more holistic predictive framework.

The general form of the multiple linear regression equation is provided as follows in Equation 3:

$$PC=P+Q\cdot TI_{1}+S\cdot TI_{2},$$ (3)

where PC represents the physicochemical property, $TI_{1}$ and $TI_{2}$ are the selected topological indices, and P,Q,S denote the intercept and regression coefficients, respectively. All possible pairwise combinations of the 16 topological indices were considered against each of the six physicochemical properties. This exhaustive modeling approach enabled the identification of synergistic relationships between the indices, leading to significantly higher $R^{2}$ values than those of single-variable models. Based on this comparative analysis, the MLR model emerged as the most robust and predictive model and was thus selected as the primary modeling framework for the study. The full list of all the 720 regression models, sorted by $R^{2}$ , is provided in the Supplementary Material (multiple_linear_regression_all_models_sorted.csv).

$$\text{MW}=6.6887-23.49\cdot \text{RLI}+24.4713\cdot \text{ISLI}\;\left(\text{p}=4.596\times 10^{-15},R^{2}=0.996\right).$$ (4)

$$\text{MW}=-0.6079-1.1\cdot \text{RLI}+21.6163\cdot \text{ISI}\;\left(\text{p}=6.055\times 10^{-17}0,R^{2}=0.998\right).$$ (5)

$$\text{MW}=3.5693+0\cdot \text{SLI}+20.1801\cdot \text{ISI}\;\left(\text{p}=2.275\times 10^{-16},R^{2}=0.9975\right).$$ (6)

$$\text{MW}=0.8916-0.72\cdot \text{ISLI}+21.6683\cdot \text{ISI}\;\left(\text{p}=1.379\times 10^{-16},R^{2}=0.9977\right).$$ (7)

$$\text{MW}=4.1857-0.35\cdot \text{MLI}+20.3502\cdot \text{ISI}\;\left(\text{p}=2.118\times 10^{-16},R^{2}=0.9976\right).$$ (8)

$$\text{MW}=-0.0239-1.12\cdot \text{MLSI}+21.8625\cdot \text{ISI}\;\left(\text{p}=1.006\times 10^{-18},R^{2}=0.999\right).$$ (9)

$$\text{MW}=5.3026-1.6\cdot \text{MII}+20.5819\cdot \text{ISI}\;\left(\text{p}=1.393\times 10^{-16},R^{2}=0.9977\right).$$ (10)

$$\text{MW}=4.7843-1.81\cdot \text{MIRI}+20.4961\cdot \text{ISI}\;\left(\text{p}=1.766\times 10^{-16},R^{2}=0.9976\right).$$ (11)

$$\text{MW}=3.6384-0.04\cdot \text{MRI}+20.183\cdot \text{ISI}\;\left(\text{p}=2.273\times 10^{-16},R^{2}=0.9975\right).$$ (12)

$$\text{MW}=3.246+0.11\cdot \text{MDI}+20.0477\cdot \text{ISI}\;\left(\text{p}=2.191\times 10^{-16},R^{2}=0.9975\right).$$ (13)

$$\text{MW}=4.6953-2.01\cdot \text{MHI}+20.5079\cdot \text{ISI}\;\left(\text{p}=1.799\times 10^{-16},R^{2}=0.9976\right).$$ (14)

$$\text{MW}=5.1032+0.61\cdot \text{MMRI}+19.2445\cdot \text{ISI}\;\left(\text{p}=1.969\times 10^{-16},R^{2}=0.9976\right).$$ (15)

$$\text{MW}=3.6144+0.03\cdot \text{MMRDI}+20.0973\cdot \text{ISI}\;\left(\text{p}=2.273\times 10^{-16},R^{2}=0.9975\right).$$ (16)

$$\text{MW}=21.28+12\cdot \text{MMDI}-2.4459\cdot \text{MMSDI}\;\left(\text{p}=8.876\times 10^{-13},R^{2}=0.9902\right).$$ (17)

$$\text{MW}=3.8245-0.13\cdot \text{MMDI}+20.4893\cdot \text{ISI}\;\left(\text{p}=2.183\times 10^{-16},R^{2}=0.9975\right).$$ (18)

$$\text{MW}=4.0969-0.03\cdot \text{MMSDI}+20.2955\cdot \text{ISI}\;\left(\text{p}=2.146\times 10^{-16},R^{2}=0.9976\right).$$ (19)

$$\text{MW}=3.551-0.04\cdot \text{SDDI}+20.332\cdot \text{ISI}\;\left(\text{p}=2.267\times 10^{-16},R^{2}=0.9975\right).$$ (20)

Here, MW denotes the molecular weight, TPSA denotes the topological polar surface area, and HBA denotes the hydrogen bond acceptor count. Of all the permutations and combinations of multiple linear regression equations, those with $R^{2}>$ 0.99 have been included in the study. Linear and quadratic regression models were formulated as well, but the multiple linear regression results were included as they yielded the best results. The multiple linear regression models were tested for all permutations and combinations of topological indices in pairs while considering each physicochemical property mentioned previously. The entries showcasing the best results with a threshold of $R^{2}=0.99$ are displayed here. The following figures represent the graphical view of the acquired equations. The plane equations represent the multi-linear models that were formulated. Each regression equation considers two indices as the independent pair of variables.

**FIGURE 5:**
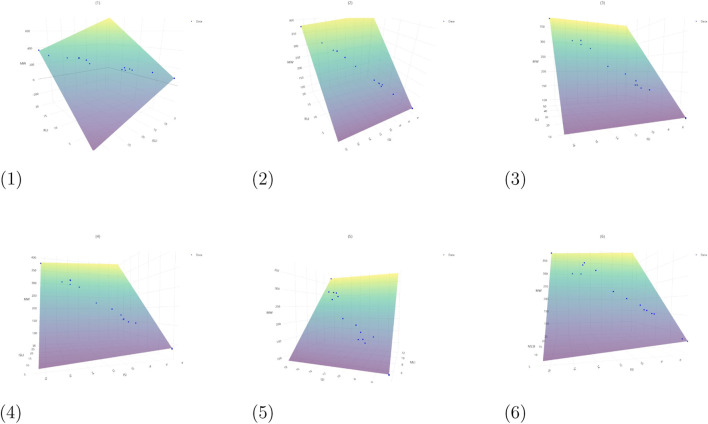
Regression Equations 4–9 for chemicals in Aegle marmelos. Graphical representation of QSPR regression equations. (1) Regression Equation 4. (2) Regression Equation 5. (3) Regression Equation 6. (4) Regression Equation 7. (5) Regression Equation 8. (6) Regression Equation 9.

**FIGURE 6:**
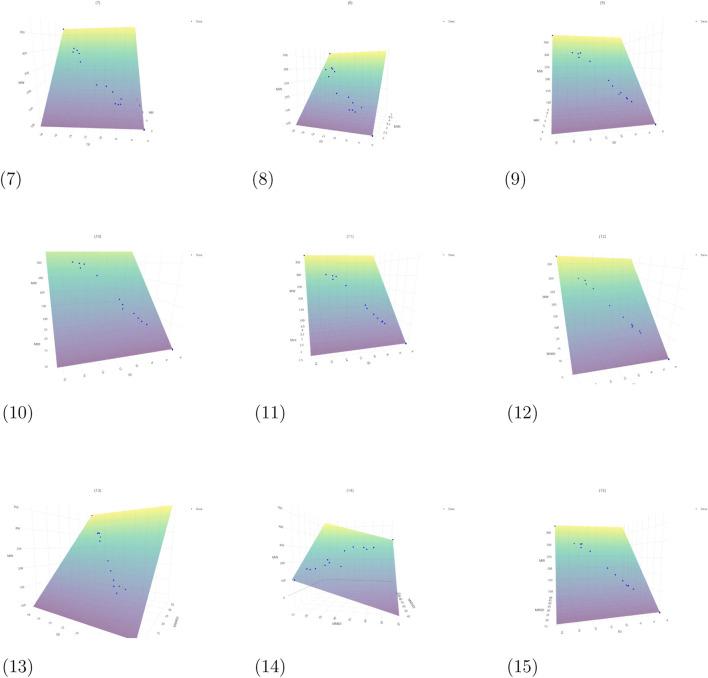
Regression Equations 10–18 for chemicals in Aegle marmelos. Graphical representation of QSPR regression equations using two topological indices. (7) Regression Equation 10. (8) Regression Equation 11. (9) Regression Equation 12. (10) Regression Equation 13. (11) Regression Equation 14. (12) Regression Equation 15. (13) Regression Equation 16. (14) Regression Equation 17. (15) Regression Equation 18.

**FIGURE 7:**
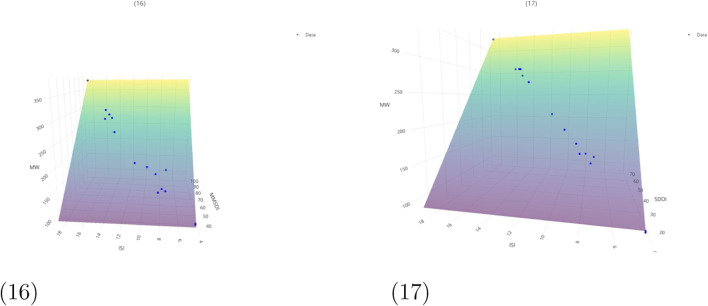
Regression Equations 19 and 20 for chemicals in Aegle marmelos. Additional QSPR regression equations showing bivariate topological relationships. (16) Regression Equation 19. (17) Regression Equation 20.

The corresponding *p*-values for the models listed in Table 6 are incorporated within the regression equations presented in Figures 5–7. Figure 5 illustrates the planar representations of regression Equations 4–9, while Figure 6 presents Equations 10–18, which were constructed using two topological indices. Furthermore, Figure 7 displays the additional bivariate multilinear models corresponding to Equations 19, 20. Each graphical representation includes the associated $R^{2}$ values, *p*-values, and regression structure, thereby visualizing the multivariate QSPR relationships of the analyzed *A. marmelos* compounds.

**TABLE 6:** Multiple linear regression results from the topological indices.

Property	$TI_{1}$	$TI_{2}$	P	Q	S	$R^{2}$	F−Statistic	p -value
MW	RLI	ISLI	6.68857	−23.4901	24.47126	0.995922	1,465.481	$4.60\times 10^{-15}$
MW	RLI	ISI	−0.60789	−1.09636	21.61635	0.998018	606.048	$8.88\times 10^{-13}$
MW	SLI	ISI	3.56930	−0.00402	20.18014	0.997529	3,021.843	$6.05\times 10^{-17}$
MW	ISLI	ISI	0.89160	−0.71872	21.66835	0.997727	2,422.487	$2.27\times 10^{-16}$
MW	MLI	ISI	4.18574	−0.35178	20.35019	0.997558	2,633.627	$1.38\times 10^{-16}$
MW	MLSI	ISI	−0.02395	−1.12036	21.86249	0.998999	2,451.440	$2.12\times 10^{-16}$
MW	MII	ISI	5.30257	−1.60489	20.58187	0.997723	5,987.795	$1.01\times 10^{-18}$
MW	MIRI	ISI	4.78427	−1.81216	20.49605	0.997631	2,629.415	$1.39\times 10^{-16}$
MW	MRI	ISI	3.63841	−0.04092	20.18296	0.997530	2,527.055	$1.77\times 10^{-16}$
MW	MDI	ISI	3.24598	0.10745	20.04767	0.997545	2,422.688	$2.27\times 10^{-16}$
MW	MHI	ISI	4.69532	−2.00541	20.50795	0.997624	2,437.658	$2.19\times 10^{-16}$
MW	MMRI	ISI	5.10319	0.61149	19.24452	0.997588	2,519.202	$1.80\times 10^{-16}$
MW	MMRDI	ISI	3.61441	0.03444	20.09729	0.997530	2,481.671	$1.97\times 10^{-16}$
MW	MMDI	MMSDI	21.28003	12.00175	−2.44589	0.990197	2,422.678	$2.27\times 10^{-16}$
MW	MMDI	ISI	3.82452	−0.13095	20.48932	0.997546	2,439.173	$2.18\times 10^{-16}$
MW	MMSDI	ISI	4.09695	−0.03303	20.29550	0.997553	2,446.080	$2.15\times 10^{-16}$
MW	SDDI	ISI	3.55097	−0.04332	20.33203	0.997531	2,423.899	$2.27\times 10^{-16}$

### 4.5 Justification for including all the physicochemical properties

This study incorporates a comprehensive set of physicochemical properties to enable robust QSPR modeling of *A. marmelos* compounds. Each descriptor—molecular weight, melting point, TPSA, hydrogen bond donor/acceptor counts, and the number of rotatable bonds—has been validated in prior studies for its relevance to drug-likeness and ADME characteristics. The melting point, for example, correlates with solubility and absorption potential via the general solubility equation (Chu and Yalkowsky, 2009), while the MW influences the release kinetics, solubility, and permeability (Toews and Bates, 2023; Shino and Kaneko, 2025). TPSA serves as a reliable surrogate for membrane permeability and brain penetration (Prasanna and Doerksen, 2009). H-bond donors and acceptors underpin Lipinski’s and Veber’s rules, reflecting their significance in oral bioavailability and solvent interaction models (Coimbra et al., 2021; Balali et al., 2021; Mercader et al., 2010). The number of rotatable bonds indicates molecular flexibility and affects bioavailability, making it a staple in QSPR/QSAR frameworks (Caron et al., 2020). These descriptors collectively ensure that the selected features balance biological relevance with predictive accuracy.

#### 4.5.1 Structural interpretations

In addition to pharmacokinetic relevance, each physicochemical property reflects molecular-level structural traits.

- MW is determined by the atomic composition and size of the molecule. Larger or more complex molecules with multiple rings or heteroatoms will naturally exhibit higher MW.
- MP is influenced by the molecular symmetry, rigidity, and intermolecular forces such as hydrogen bonding. Highly crystalline or symmetrical molecules often have higher MP due to efficient packing.
- TPSA reflects the contribution of polar atoms (e.g., O and N) and functional groups such as hydroxyl and carboxyl, which affect the hydrogen bonding capacity and membrane permeability.
- HBD and HBA counts are directly associated with the presence of groups such as –OH, –NH (donors), and = O, ether, or nitro groups (acceptors), thus influencing solubility and binding interactions.
- RBC captures molecular flexibility by quantifying the single, non-ring, and non-terminal bonds. Molecules with high RBC are more conformationally dynamic but may face reduced bioavailability due to entropic penalties upon binding.

## 5 Ranking of compounds

Ranking methods and MCDM techniques play a crucial role in evaluating the physicochemical properties and bioactivity of drug compounds. These methods support the comparison of multiple drug candidates by incorporating diverse evaluation criteria such as efficacy, toxicity, and solubility. Techniques such as TOPSIS (Hwang and Yoon, 1981), VIKOR (Opricovic and Tzeng, 2004), and SAW (Churchman et al., 1957) enable structured decision-making through the assignment of weights and the prioritization of alternatives. MCDM approaches are particularly useful in balancing conflicting objectives—such as maximizing the therapeutic effect while minimizing adverse effects. Additionally, advanced variations such as fuzzy MCDM and Bayesian ranking can address uncertainty in experimental datasets. These frameworks can also be integrated with AI/ML models to refine or validate predictions, thereby contributing to more reliable drug screening and selection processes.

This study utilizes VIKOR, SAW, and TOPSIS to rank *A. marmelos* compounds and assess the consistency of their prioritizations. These MCDM techniques were selected for their broad adoption, interpretability, and complementary decision logic. For instance, VIKOR has been used effectively in QSPR-based drug evaluations (Ashraf and Idrees, 2024), while TOPSIS has been applied in therapeutic compound screening using SMAA extensions (Peng et al., 2021). SAW, known for its simplicity and transparency, serves as a baseline additive method. Comparing these approaches offers a multi-angle perspective on compound ranking. The following sections outline their implementation.

### 5.1 Methodology for assigning weights

The data used for assigning weights to topological indices is derived from the MLR model results. Specifically, only those regression equations satisfying the threshold condition $R^{2}\geq 0.99$ are considered for further analysis. As provided in Table 6, we observe that all selected models conform to this form. The total frequency of occurrence of any TI, as indicated in Table 6, is denoted as $f_{TI_{i}}$ , where $TI_{i}$ represents a specific type of discrete Adriatic topological index.

The weight of the respective topological index value is calculated as follows in Equation 21:

$$w_{TI_{i}}=\frac{f_{TI_{i}}}{\sum_{i=1}^{k}f_{TI_{i}}}.$$ (21)

Here, $\sum_{i=1}^{k}f_{TI_{i}}=34.$

**FIGURE 8:**
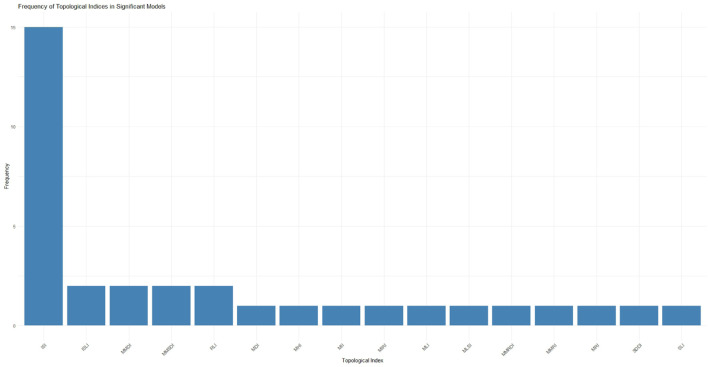
Frequency distribution of the topological indices based on their appearance in statistically significant QSPR regression models.

Figure 8 presents the frequency distribution of topological indices based on their occurrences across statistically significant QSPR regression models. This analysis highlights the relative importance of each index in capturing relevant structural information associated with the physicochemical properties of *A. marmelos* compounds. The ISI index, appearing most frequently, indicates a consistent contribution to model performance and is, therefore, assigned a higher weight in subsequent MCDM-based ranking. Indices with lower frequencies were included to preserve structural diversity, but their limited presence may indicate weaker predictive utility. The calculated weights were utilized in the VIKOR, SAW, and TOPSIS MCDM ranking schemes. Specifically, the frequency with which each topological index appeared in statistically significant regression models (see Table 7) was used to derive its weight for the MCDM analysis. These frequency-based weights (summarized in Table 8) ensure that the prioritization reflects the relative predictive influence of each descriptor observed in the QSPR modeling.

**TABLE 7:** Assigned $f_{TI_{i}}$ values.

RLI	SLI	ISLI	MLI	MLSI	MII	MIRI	MRI	MDI	MHI	MMRI	MMRDI	MMDI	MMSDI	SDDI	ISI
2	1	2	1	1	1	1	1	1	1	1	1	2	2	1	15

**TABLE 8:** Assigned criterion weights.

RLI	SLI	ISLI	MLI	MLSI	MII	MIRI	MRI	MDI	MHI	MMRI	MMRDI	MMDI	MMSDI	SDDI	ISI
0.059	0.029	0.059	0.029	0.029	0.029	0.029	0.029	0.029	0.029	0.029	0.029	0.059	0.059	0.029	0.441

### 5.2 Justification for the MCDM method selection

In this study, three widely used multi-criteria decision-making methods, namely, SAW, TOPSIS, and VIKOR, were utilized for compound ranking. These techniques were selected based on their complementary methodological foundations.

SAW offers a transparent and additive scoring framework that is particularly suitable for initial prioritization tasks. TOPSIS identifies the alternative that is geometrically closest to the ideal solution, making it advantageous for situations requiring spatial interpretability. VIKOR, in contrast, emphasizes compromise ranking and is suitable when there is a need to balance conflicting criteria. Although other MCDM methods, such as WASPAS, MORA, AHP, and PROMETHEE I and II, are available, the selected trio of SAW, TOPSIS, and VIKOR represents a diverse range of decision strategies, including additive, distance-based, and compromise models. This selection enables robust cross-validation of the results. The high level of agreement observed across the rankings produced by these three methods (see Figure 10) further reinforces the consistency and reliability of the adopted prioritization framework. Although SAW, TOPSIS, and VIKOR were selected to represent a diverse spectrum of decision-making strategies, this study did not perform a comparative analysis with other widely used MCDM methods such as PROMETHEE, AHP, or WASPAS. Such comparative benchmarking was beyond the scope of the current work but remains an important future direction to assess the generalizability and sensitivity of compound rankings under alternative mathematical frameworks.

### 5.3 VIKOR ranking

The chemicals in *A. marmelos* were analyzed using the VIKOR method, which is a well-established MCDM technique. This approach is particularly useful in scenarios involving conflicting criteria, where a compromise solution is preferable to a purely optimal one. By applying VIKOR, the study ranks the chemical compounds based on multiple physicochemical and biological attributes, thereby helping identify the most balanced candidates for further studies in drug development.

#### 5.3.1 Criterions and decision matrix

The physicochemical compounds that need to be ranked are termed as alternatives $\left(\alpha_{m}\right)$ . The set of all topological indices from the regression models was considered as criteria $\left(\beta_{n}\right)$ . However, the weights were assigned based on their fit. A decision matrix is constructed, γ = $\left[\theta_{mn}\right]$ , where $\theta_{mn}$ represents the performance of $\alpha_{m}$ under the criterion $\beta_{n}$ .

#### 5.3.2 Criteria weights

Weights were assigned based on the relative importance of TIs, as determined by the fit of the model. It was ensured that $\sum_{i=1}^{n}w_{TI_{i}}=1$ . The weights were assigned based on the relative contribution of each topological index in the multiple linear regression models, as indicated by their $R^{2}$ values and frequency of occurrence across the best-fitting models. This regression-driven weighting aligns the MCDM evaluation with the actual predictive influence of each index. Although alternative methods such as Entropy, COPRAS, or AHP are widely accepted, our approach was chosen to maintain coherence between statistical modeling and decision-making. Future work could compare the results from such methods to validate the robustness of the ranking framework.

#### 5.3.3 Best (maximum) and worst (minimum) results

The maximum and minimum of the alternatives are identified. The beneficial criteria are calculated as ${\text{x}}_{n}^{*}=max_{n}\left(\theta_{mn}\right)$ , ${\text{x}}_{n}^{-}=min_{n}\left(\theta_{mn}\right)$ .The non-beneficial criteria are calculated as ${\text{x}}_{n}^{*}=min_{n}\left(\theta_{mn}\right)$ and ${\text{x}}_{n}^{-}=max_{n}\left(\theta_{mn}\right)$ . Here, n denotes the maximum or minimum value along the particular column confined to column n.

Utility and regret measure: the utility measure ${\text{S}}_{i}$ and the regret measure ${\text{R}}_{i}$ are computed.

Utility measure: ${\text{S}}_{i}=\sum_{n=1}^{k}\left[w_{n}\times \frac{x_{n}^{*}-\theta_{mn}}{x_{n}^{*}-x_{n}^{-}}\right]$ . Regret measure: $R_{i}=max\left[w_{n}\times \frac{x_{n}^{*}-\theta_{mn}}{x_{n}^{*}-x_{n}^{-}}\right]$ .

#### 5.3.4 VIKOR index

The parameter λ serves to balance between the majority decision and individual dominance, while the final VIKOR ranking is determined based on the VIKOR index $\left(\Omega_{m}\right)$ as shown in Equation 22.

$$\Omega_{m}=\lambda \frac{S_{i}-S^{*}}{S^{-}-S^{*}}+\left(1-\lambda \right)\frac{R_{i}-R^{*}}{R^{-}-R^{*}},$$ (22)

where λ is the weight of the strategy. Here, λ=0.5 , where $S^{*}=min_{i}\left(S_{i}\right)$ , $S^{-}=max_{i}\left(S_{i}\right),R^{*}=min_{i}\left(R_{i}\right)$ , and $R^{-}=max_{i}\left(R_{i}\right)$ .

#### 5.3.5 VIKOR ranking

The alternatives $\Omega_{m}$ are ordered in ascending sequence, with the one having the smallest value assigned the top rank (best alternative), followed by the others accordingly.

### 5.4 SAW ranking

The SAW ranking refers to the SAW method—a popular technique in MCDM. It is used to rank alternatives based on multiple attributes (criteria) and their assigned importance (weights). SAW is intuitive, easy to implement, and provides a straightforward linear scoring model. In other words, the SAW method, a classical form of the multi-attribute value approach, constructs a value function by summing the scores that indicate goal achievement for each criterion, each multiplied by its respective weight (Taherdoost, 2023).

#### 5.4.1 Alternatives, criterions, and decision matrix

A decision matrix $\gamma =\left[\theta_{mn}\right]$ is defined using the criteria $\beta_{n}$ that is used to evaluate the alternatives $\alpha_{m}$ .

#### 5.4.2 Normalization, weights, and weighted score evaluation

The decision matrix is normalized to ensure that all criteria are on the same scale using $\theta_{mn}^{*}=\frac{\theta_{mn}}{\theta_{m}^{*}}$ , where $\theta_{m}^{*}=max_{m}\left(\theta_{mn}\right)$ for the beneficial criteria, and $\theta_{mn}^{*}=\frac{\theta_{m}^{*}}{\theta_{mn}}$ , where $\theta_{m}^{*}=min_{m}\left(\theta_{mn}\right)$ for the non-beneficial criteria. The weights are assigned to each criterion based on their relative importance, which was derived from the regression analysis. The weights satisfy the equation $\sum_{n=1}^{k}w_{n}=1$ . The weighted scores are then calculated as follows: $A_{mn}=w_{n}\times \theta_{mn}$ .

#### 5.4.3 Evaluation of the total score and ranking system

The total scores are evaluated as $TS_{m}=\sum_{n=1}^{k}A_{mn}$ . The evaluation of the alternatives was conducted using a hierarchical ranking system. Here, the higher numerical values corresponded to enhanced effectiveness.

### 5.5 TOPSIS ranking

TOPSIS is an MCDM method that ranks alternatives based on their proximity to the ideal solution and their distance from the negative ideal solution. It serves as an effective MADM approach, enabling the analysis, comparison, and ranking of alternatives to identify the most appropriate and optimal option according to the problem’s criteria (Madanchian and Taherdoost, 2023).

#### 5.5.1 Alternatives, criteria, and decision matrix

A decision matrix $\gamma =\left[\theta_{mn}\right]$ is defined using the criteria $\beta_{n}$ that is used to evaluate the alternatives $\alpha_{m}$ .

#### 5.5.2 Normalization and assignment of weights

The decision matrix is normalized to ensure that all criteria are on the same scale using $\theta_{mn}^{*}=\frac{\theta_{mn}}{\sqrt{\sum_{m=1}^{k}\theta_{mn}^{2}}}$ . The weights are assigned to each criterion on the basis of their relative importance, which were derived from the regression analysis. The weights satisfy the equation $\sum_{n=1}^{k}w_{n}=1$ .

#### 5.5.3 Calculation of weighted sums and determination of the ideal solutions

The weighted sums are calculated as $v_{mn}=w_{n}\times \theta_{mn}$ . The positive and negative ideal solutions are to be decided for the beneficial and non-beneficial criteria. For the positive ideal solution, $I^{+}=\left\{v_{1}^{*},\ldots ,v_{k}^{*}\right\}$ , where $v_{n}^{*}=\left\{max_{m}\left(v_{mn}\right)\;if\;n\in Beneficial;\;min_{m}\left(v_{mn}\right)\;n\in Non-Beneficial\;\right\}$ . For the negative ideal solution, $I^{-}=\left\{v_{1}^{\prime },\ldots ,v_{k}^{\prime }\right\}$ , where $v_{n}^{\prime }=\left\{min_{m}\left(v_{mn}\right)\;if\;n\in Beneficial;\;max_{m}\left(v_{mn}\right)\;n\in Non-Beneficial\right\}$ .

### 5.6 Calculation of the separation measures for each alternative

The separation from the positive ideal solution is calculated as $S_{m}^{+}=\sqrt{\sum_{n=1}^{k}{\left(v_{n}^{*}-v_{mn}\right)}^{2}}$ , and the separation from the negative ideal solution is calculated as $S_{m}^{\prime }=\sqrt{\sum_{n=1}^{k}{\left(v_{n}^{\prime }-v_{mn}\right)}^{2}}$ .

#### 5.6.1 Calculation of the relative closeness to the ideal solution and ranking

The relative closeness to the ideal solution is determined using $C_{m}^{*}=\frac{S_{m}^{-}}{S_{m}^{-}+S_{m}^{+}}$ . The alternatives were assessed using a hierarchical ranking system, with higher values indicating greater effectiveness.

## 6 Materials and methods

This study analyzed 15 pharmacologically relevant compounds (auraptene, aegeline, kaempferol, ferulic acid, ellagic acid, umbelliferone, 1,8-cineole, acetoin, citronellal, eugenol, oxalic acid, tembamide, riboflavin, xanthotoxin, and ascorbic acid) that represent the major and most frequently reported bioactive constituents of *A. marmelos* with reliable physicochemical data available in public databases. Structural information was sourced from corresponding .mol files and parsed using Python scripts to compute 16 discrete Adriatic TIs, which quantify the atomic connectivity and bonding patterns.

For statistical analysis, RStudio (version 4.4.2) was used to construct 720 total MLR models derived from 120 pairwise combinations for each of the six physicochemical properties. The model quality was evaluated using $R^{2}$ , p -values, and F-statistics. Specifically, the average $R^{2}$ across all 120 models was used for the general property predictability ranking, while a strict $R^{2}\geq 0.99$ filter was applied to select the most statistically robust models for the subsequent analysis.

The final phase involved compound prioritization using three MCDM techniques—SAW, TOPSIS, and VIKOR—which were implemented via custom R scripts. The criterion weights for the MCDM analysis were derived objectively from the QSPR results, which were calculated by normalizing the frequency of occurrence of each TI within the highly predictive $R^{2}\geq 0.99$ regression models. This methodology established a unified, data-driven framework linking the structural descriptors to compound ranking.

## 7 Results

The molecular structures of the bioactive physicochemicals present in *A. marmelos* were studied using degree-based discrete Adriatic topological indices. The $R^{2}$ value determines the extent of variance of a dependent variable with respect to an independent variable. It is used to quantify the extent to which the observed data can be predicted. The *p*-value or probability value has also been provided. Simple linear regression models, quadratic regression models, and multiple linear regression models were tested. However, the multiple linear regression results were included as they yielded the best results.

### 7.1 Multiple linear regression

To assess the combined predictive capability of molecular topological indices on selected physical properties, multiple linear regression analyses were conducted using pairs of descriptors from the computed index set. Each physical property (melting point, molecular weight, TPSA, HBD, HBA, and RBC) was regressed against all possible pairs of topological indices, and the coefficient of determination (R^2^) was used to evaluate the model performance.

The threshold for significance was set at R^2^ > 0.99, which is in accordance with the rigor required for predictive modeling in cheminformatics. Among all combinations, significant models were obtained only for molecular weight, with 17 pairs of topological indices surpassing the threshold (Table 6). The highest R^2^ value (0.9989) was achieved using MLSI and ISI, indicating an exceptionally strong linear relationship. Other notable high-performing pairs included RLI and ISI, SLI and ISI, and ISLI and ISI, all with R^2^ values above 0.99. No pairwise models for melting point, TPSA, HBDs, HBAs, or RBC met the established R^2^ criterion.

These results indicate that while single or paired indices may not be sufficient to explain more complex properties such as melting point or TPSA, certain descriptors (notably ISI) have exceptional predictive power for molecular weight when combined appropriately. It has also been observed that the ISI topological index is of great importance for predicting the physicochemical properties. The ability of the regression model to accurately predict the molecular weight, in turn, assists in predicting other properties that help determine the ADMET profile of chemical compounds.

### 7.2 VIKOR ranking of Aegle marmelos chemicals

The multiple regression model fits were used to rank compounds. The regression models played an important role in determining the weights of each of the TIs values. All details have been included in Section 5.1. The necessary topological indices along with their respective weights are presented in Table 9. Some important details regarding the normalized matrix are in Table 10. The rankings, along with the calculated utility measures, regret measures, and VIKOR indices, are provided in Table 11.

**TABLE 9:** Assigned criterion weights.

RLI	SLI	ISLI	MLI	MLSI	MII	MIRI	MRI	MDI	MHI	MMRI	MMRDI	MMDI	MMSDI	SDDI	ISI
0.059	0.029	0.059	0.029	0.029	0.029	0.029	0.029	0.029	0.029	0.029	0.029	0.059	0.059	0.029	0.441

**TABLE 10:** VIKOR normalized matrix for *Aegle marmelos* chemicals.

Molecule	RLI	SLI	ISLI	MLI	MLSI	MII	MIRI	MRI	MDI	MHI	MMRI	MMRDI	MMDI	MMSDI	SDDI	ISI
Aegeline	0.020	0.007	0.018	0.020	0.017	0.023	0.021	0.019	0.018	0.021	0.005	0.010	0.027	0.040	0.010	0.135
Auraptene	0.020	0.007	0.017	0.014	0.015	0.017	0.015	0.013	0.012	0.015	0.006	0.009	0.021	0.028	0.009	0.133
Ellagic acid	0.001	0.003	0.006	0.012	0.000	0.013	0.012	0.012	0.012	0.012	0.003	0.006	0.015	0.018	0.006	0.104
Ferulic acid	0.039	0.018	0.038	0.022	0.021	0.023	0.022	0.021	0.021	0.022	0.018	0.019	0.041	0.045	0.020	0.282
Kaempferol	0.012	0.006	0.014	0.013	0.008	0.015	0.014	0.012	0.012	0.013	0.006	0.009	0.020	0.024	0.008	0.137
Umbelliferone	0.036	0.018	0.038	0.026	0.025	0.029	0.028	0.025	0.024	0.027	0.019	0.021	0.046	0.054	0.022	0.332
1,8-Cineole	0.038	0.019	0.040	0.018	0.025	0.023	0.021	0.015	0.012	0.021	0.021	0.020	0.038	0.027	0.020	0.350
Acetoin	0.059	0.029	0.059	0.029	0.029	0.029	0.029	0.029	0.029	0.029	0.029	0.029	0.059	0.056	0.029	0.441
Citronellal	0.051	0.024	0.049	0.027	0.027	0.027	0.027	0.027	0.026	0.027	0.023	0.024	0.050	0.053	0.024	0.344
Eugenol	0.041	0.020	0.042	0.028	0.025	0.029	0.029	0.027	0.026	0.028	0.020	0.022	0.049	0.059	0.023	0.320
Oxalic acid	0.059	0.029	0.059	0.029	0.028	0.029	0.029	0.029	0.029	0.029	0.029	0.029	0.059	0.056	0.029	0.441
Tembamide	0.022	0.009	0.021	0.023	0.020	0.025	0.024	0.022	0.021	0.023	0.008	0.013	0.031	0.044	0.013	0.171
Riboflavin	0.000	0.000	0.000	0.000	0.000	0.000	0.000	0.000	0.000	0.000	0.000	0.000	0.000	0.000	0.000	0.000
Xanthotoxin	0.021	0.012	0.025	0.026	0.018	0.029	0.028	0.025	0.024	0.027	0.011	0.016	0.039	0.053	0.016	0.245
Ascorbic acid	0.040	0.021	0.041	0.023	0.025	0.023	0.023	0.023	0.024	0.023	0.020	0.021	0.044	0.044	0.021	0.311

**TABLE 11:** VIKOR summary of the decision metrics and rankings.

Molecule	$S_{i}$	$R_{i}$	Qi	Rank
Aegeline	0.410	0.135	0.358	5
Auraptene	0.352	0.133	0.327	4
Ellagic acid	0.235	0.104	0.236	2
Ferulic acid	0.672	0.282	0.656	8
Kaempferol	0.323	0.137	0.317	3
Umbelliferone	0.770	0.332	0.762	12
1,8-Cineole	0.707	0.350	0.751	10
Acetoin	0.997	0.441	1.000	15
Citronellal	0.829	0.344	0.805	13
Eugenol	0.790	0.320	0.758	11
Oxalic acid	0.995	0.441	0.999	14
Tembamide	0.489	0.171	0.439	6
Riboflavin	0.000	0.000	0.000	1
Xanthotoxin	0.616	0.245	0.586	7
Ascorbic acid	0.729	0.311	0.717	9

### 7.3 SAW ranking of Aegle marmelos chemicals

The multiple regression model fits were used to rank compounds based on the SAW ranking. The regression models played an important role in determining the weights of each of the TIs values. All details have been included in Section 5.2. The necessary topological indices, along with their respective weights, are presented in Table 12. Some important details regarding the normalized matrix are provided in Table 13. The rankings, along with the total scores, are provided in Table 14.

**TABLE 12:** Assigned criterion weights (SAW method).

RLI	SLI	ISLI	MLI	MLSI	MII	MIRI	MRI	MDI	MHI	MMRI	MMRDI	MMDI	MMSDI	SDDI	ISI
0.059	0.029	0.059	0.029	0.029	0.029	0.029	0.029	0.029	0.029	0.029	0.029	0.059	0.059	0.029	0.441

**TABLE 13:** SAW normalized matrix for *Aegle marmelos* chemicals.

Molecule	RLI	SLI	ISLI	MLI	MLSI	MII	MIRI	MRI	MDI	MHI	MMRI	MMRDI	MMDI	MMSDI	SDDI	ISI
Aegeline	0.687	0.790	0.742	0.538	0.519	0.500	0.520	0.556	0.571	0.528	0.849	0.730	0.663	0.535	0.729	0.765
Auraptene	0.676	0.787	0.743	0.678	0.595	0.636	0.658	0.697	0.714	0.667	0.818	0.764	0.733	0.671	0.762	0.767
Ellagic acid	0.990	0.899	0.913	0.720	1.000	0.727	0.724	0.717	0.714	0.722	0.899	0.833	0.812	0.796	0.848	0.818
Ferulic acid	0.383	0.457	0.442	0.500	0.409	0.500	0.500	0.500	0.500	0.500	0.477	0.485	0.485	0.477	0.481	0.509
Kaempferol	0.809	0.809	0.798	0.699	0.788	0.682	0.691	0.707	0.714	0.694	0.815	0.772	0.752	0.724	0.776	0.760
Umbelliferone	0.422	0.457	0.436	0.398	0.303	0.364	0.381	0.414	0.429	0.389	0.457	0.434	0.416	0.374	0.429	0.421
1,8-Cineole	0.401	0.423	0.413	0.588	0.300	0.500	0.540	0.646	0.714	0.528	0.378	0.461	0.521	0.693	0.474	0.390
Acetoin	0.062	0.127	0.134	0.322	0.181	0.364	0.342	0.303	0.286	0.333	0.142	0.212	0.257	0.355	0.219	0.231
Citronellal	0.191	0.296	0.284	0.381	0.253	0.409	0.395	0.369	0.357	0.389	0.338	0.355	0.366	0.391	0.357	0.401
Eugenol	0.345	0.393	0.381	0.360	0.297	0.364	0.362	0.359	0.357	0.361	0.423	0.398	0.376	0.321	0.390	0.442
Oxalic acid	0.062	0.127	0.134	0.322	0.226	0.364	0.342	0.303	0.286	0.333	0.142	0.212	0.257	0.355	0.219	0.231
Tembamide	0.646	0.723	0.685	0.479	0.450	0.455	0.467	0.490	0.500	0.472	0.779	0.665	0.604	0.492	0.667	0.701
Riboflavin	1.000	1.000	1.000	1.000	0.987	1.000	1.000	1.000	1.000	1.000	1.000	1.000	1.000	1.000	1.000	1.000
Xanthotoxin	0.671	0.652	0.633	0.398	0.496	0.364	0.381	0.414	0.429	0.389	0.669	0.565	0.505	0.386	0.562	0.572
Ascorbic acid	0.364	0.386	0.396	0.462	0.316	0.500	0.480	0.444	0.429	0.472	0.405	0.428	0.446	0.487	0.433	0.458

**TABLE 14:** SAW ranking system for *Aegle marmelos* chemicals.

Molecule	$TS_{i}$	Rank
Aegeline	0.69300	5
Auraptene	0.73327	4
Ellagic acid	0.82644	2
Ferulic acid	0.48570	8
Kaempferol	0.75655	3
Umbelliferone	0.41385	11
1,8-Cineole	0.45458	9
Acetoin	0.23258	15
Citronellal	0.36396	13
Eugenol	0.39829	12
Oxalic acid	0.23391	14
Tembamide	0.63299	6
Riboflavin	0.99962	1
Xanthotoxin	0.53807	7
Ascorbic acid	0.44140	10

### 7.4 TOPSIS ranking of Aegle marmelos chemicals

The multiple regression model fits were used to rank compounds based on the TOPSIS ranking. The regression models played an important role in determining the weights of each of the topological index values. All details have been included in Section 5.3. The necessary topological indices, along with their respective weights, are provided in Table 15. Some important details regarding the normalized matrix are provided in Table 16. The rankings, along with the total scores, are provided in Table 17.

**TABLE 15:** Assigned criterion weights (TOPSIS method).

RLI	SLI	ISLI	MLI	MLSI	MII	MIRI	MRI	MDI	MHI	MMRI	MMRDI	MMDI	MMSDI	SDDI	ISI
0.059	0.029	0.059	0.029	0.029	0.029	0.029	0.029	0.029	0.029	0.029	0.029	0.059	0.059	0.029	0.441

**TABLE 16:** TOPSIS normalized matrix for chemicals of *Aegle marmelos*.

Molecule	RLI	SLI	ISLI	MLI	MLSI	MII	MIRI	MRI	MDI	MHI	MMRI	MMRDI	MMDI	MMSDI	SDDI	ISI
Aegeline	0.301	0.332	0.319	0.251	0.249	0.237	0.245	0.256	0.260	0.249	0.347	0.315	0.293	0.242	0.314	0.326
Auraptene	0.296	0.330	0.319	0.316	0.285	0.302	0.310	0.321	0.325	0.314	0.334	0.330	0.324	0.304	0.328	0.327
Ellagic acid	0.434	0.377	0.392	0.336	0.479	0.345	0.341	0.331	0.325	0.340	0.367	0.360	0.359	0.360	0.365	0.349
Ferulic acid	0.168	0.192	0.190	0.233	0.196	0.237	0.236	0.231	0.227	0.236	0.195	0.210	0.215	0.216	0.207	0.217
Kaempferol	0.354	0.340	0.343	0.326	0.378	0.324	0.325	0.326	0.325	0.327	0.333	0.333	0.333	0.328	0.334	0.324
Umbelliferone	0.185	0.192	0.187	0.186	0.145	0.173	0.180	0.191	0.195	0.183	0.187	0.188	0.184	0.169	0.185	0.180
1,8-Cineole	0.176	0.178	0.178	0.274	0.144	0.237	0.254	0.298	0.325	0.249	0.154	0.199	0.231	0.314	0.204	0.166
Acetoin	0.027	0.053	0.058	0.150	0.087	0.173	0.161	0.140	0.130	0.157	0.058	0.091	0.114	0.161	0.094	0.098
Citronellal	0.083	0.124	0.122	0.178	0.121	0.194	0.186	0.170	0.162	0.183	0.138	0.153	0.162	0.177	0.154	0.171
Eugenol	0.151	0.165	0.164	0.168	0.142	0.173	0.170	0.165	0.162	0.170	0.173	0.172	0.166	0.145	0.168	0.188
Oxalic acid	0.027	0.053	0.058	0.150	0.108	0.173	0.161	0.140	0.130	0.157	0.058	0.091	0.114	0.161	0.094	0.098
Tembamide	0.283	0.304	0.294	0.223	0.216	0.216	0.220	0.226	0.227	0.222	0.318	0.287	0.267	0.223	0.287	0.299
Riboflavin	0.438	0.420	0.430	0.466	0.473	0.475	0.471	0.461	0.455	0.471	0.408	0.432	0.442	0.453	0.431	0.426
Xanthotoxin	0.294	0.274	0.272	0.186	0.238	0.173	0.180	0.191	0.195	0.183	0.273	0.244	0.223	0.175	0.242	0.244
Ascorbic acid	0.159	0.162	0.170	0.215	0.152	0.237	0.226	0.205	0.195	0.222	0.165	0.185	0.197	0.220	0.187	0.195

**TABLE 17:** TOPSIS ranking system for *Aegle marmelos* chemicals.

Molecule	$S^{+}$	$S^{-}$	$C^{*}$	Rank
Aegeline	0.05111	0.10510	0.67280	5
Auraptene	0.04833	0.10637	0.68758	4
Ellagic acid	0.03642	0.11894	0.76556	2
Ferulic acid	0.09945	0.05490	0.35568	8
Kaempferol	0.04811	0.10643	0.68869	3
Umbelliferone	0.11611	0.03880	0.25046	11
1,8-Cineole	0.11980	0.03601	0.23112	12
Acetoin	0.15406	0.00089	0.00577	15
Citronellal	0.12168	0.03290	0.21284	13
Eugenol	0.11365	0.04145	0.26723	10
Oxalic acid	0.15402	0.00110	0.00708	14
Tembamide	0.06335	0.09263	0.59386	6
Riboflavin	0.00018	0.15415	0.99883	1
Xanthotoxin	0.08730	0.06876	0.44060	7
Ascorbic acid	0.10919	0.04510	0.29231	9

## 8 Discussion

### 8.1 Overview of the predictive analysis

In this study, we analyzed the chemical constituents of *A. marmelos* using discrete Adriatic degree-based TIs to develop QSAR and QSPR models. A combination of correlation and MLR analyses was utilized to examine how these descriptors relate to various physicochemical properties. The predictive capacity of the TIs was examined both individually and pairwise.

### 8.2 Predictive performance of the topological indices

The findings identify ISI as the most predictive TI, closely followed by MMRDI, MMRI, and SDDI. Additionally, MMSDI and MMDI also exhibited high performance. These top-six indices demonstrated consistent reproducibility in regression models. Moderately predictive indices included MDI, MLI, and RLI, while indices such as MHI, MRI, and ISLI showed lower predictive power. The least predictive descriptors were MLSI and SLI. The consistent pre-eminence of ISI—which appeared in 15 of the 17 top MW models—is a key finding. The ISI, a degree-based index, excels at capturing the subtle structural complexity, including the rigid ring systems and the varying degrees of branching characteristic of the *A. marmelos* phytochemicals. This ability to capture the structural size and connectivity efficiently directly translates to its strong correlation with size-dependent properties such as MW and TPSA. This high statistical performance not only validates the descriptor but also inherently justifies the ISI’s high weight as a primary criterion in the subsequent compound prioritization process.

### 8.3 Property-wise predictability

**FIGURE 9:**
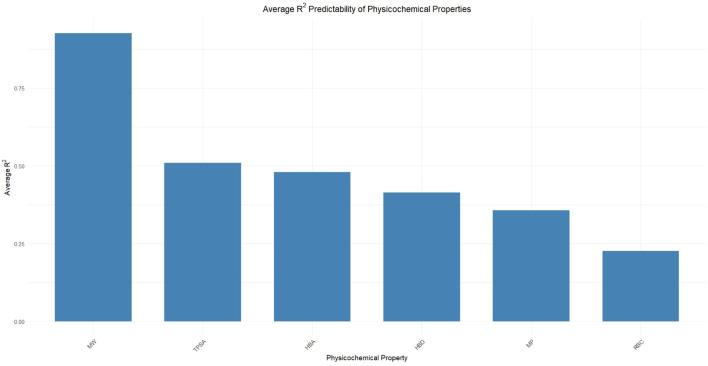
Regression-based predictability scores for the physicochemical properties, highlighting the superior performance for size- and connectivity-driven properties (MW and TPSA) compared to that of flexibility-dependent properties (RBC).

Predictive accuracy for the physicochemical properties showed a distinct descending trend, with MW being the most predictable, followed by TPSA, HBA count, HBD count, MP, and, finally, RBC, as illustrated in Figure 9. This trend reflects the strong dependence of MW and TPSA on the molecular size and connectivity, which are effectively captured by degree-based topological indices. In contrast, properties such as MP and RBC are influenced by conformational flexibility and intermolecular interactions, leading to reduced predictability using two-dimensional (2D) descriptors alone.

This order emphasizes the strong structural dependencies of MW and TPSA on topological indices and the relative difficulty of modeling properties such as RBC using 2D descriptors alone. The significant decrease in predictability for RBC and MP confirms a known limitation of 2D TIs. Properties such as RBC and MP are heavily influenced by conformational flexibility, intermolecular forces, and three-dimensional (3D) spatial arrangement, effects that the 2D graph theory cannot fully capture. In contrast, the strong success with MW and TPSA demonstrates that the Adriatic TIs are highly effective at modeling the structural size and connectivity attributes.

### 8.4 Modeling framework and statistical robustness

Each physicochemical property was modeled as a function of two distinct TIs using the following equation as shown in Equation 23.

$$PC=P+\left[Q\times TI_{1}\right]+\left[S\times TI_{2}\right],$$ (23)

where PC denotes the property of interest and P , Q , and S are the regression coefficients. Given the 16 topological indices, a total of 120 unique pairwise MLR models were developed for each of the six properties, leading to 720 total regression models for this study. The analysis proceeded in two distinct stages. First, to assess the general property predictability, the ranking of properties (as shown in Figure 9) was derived from the average $R^{2}$ value calculated across the full set of 120 models for each property, allowing a complete, full-range exploration of relationships, as documented in the Supplementary Material. Second, to ensure the objectivity of the subsequent MCDM analysis, we applied a strict statistical filter: only those models achieving $R^{2}\geq 0.99$ and having a significant p<0.05 were retained. This rigorous filtering was essential because the frequency of TIs in this highly reliable subset was used to derive the weights for the MCDM ranking.

Correlation matrix results (Table 3) showed that MW had the strongest individual linear associations with most TIs (e.g., ISI: 0.9988, SDDI: 0.9879, and ISLI: 0.9788). TPSA and HBA also demonstrated moderate correlations. In contrast, MP and HBD showed weaker and less consistent associations, and RBC showed the lowest correlations (e.g., MMSDI: 0.1416 and RLI: 0.1935).

Regression-based models reinforced these findings, with MW again showing the highest average $R^{2}$ (well above 0.8). TPSA and HBA maintained strong predictability, while HBD’s predictability slightly improved compared to its individual correlations. MP exhibited reduced performance, possibly due to the non-linear effects. RBC remained the least predictable property, reaffirming its poor dependence on topological descriptors.

### 8.5 Significant regression models and key predictors

To determine the statistical significance of each regression model, we examined the coefficient of determination $\left(R^{2}\right)$ , the overall model p -value (from the F-test), and the p -values of individual predictors. A model was considered statistically significant if the overall p -value was less than 0.05, and both predictors had p<0.05 . The best model for each property was selected based on the highest $R^{2}$ value combined with statistical significance. Table 18 summarizes the best TI pairs, $R^{2}$ , and the significance status for each physicochemical property.

**TABLE 18:** Summary of the best models for each physicochemical property.

Property	TI_1_	TI_2_	$R^{2}$	Significant (p<0.05)
MW	MLSI	ISI	0.999	Yes
TPSA	MII	ISI	0.893	Yes
HBA	MHI	ISI	0.866	Yes
HBD	MII	ISI	0.802	Yes
MP	MLSI	ISI	0.798	Yes
RBC	MMSDI	ISI	0.615	Yes

Table 19 lists the models with statistically significant p -values for MW. All 17 models had $R^{2}>0.99$ and p<0.05 , validating their robustness.

**TABLE 19:** Summary of model p -values for the multiple regression models.

Physical quantity	TI_1_	TI_2_	Model p -value
MW	RLI	ISLI	$4.596\times 10^{-15}$
MW	RLI	ISI	$6.055\times 10^{-17}$
MW	SLI	ISI	$2.275\times 10^{-16}$
MW	ISLI	ISI	$1.379\times 10^{-16}$
MW	MLI	ISI	$2.118\times 10^{-16}$
MW	MLSI	ISI	$1.006\times 10^{-18}$
MW	MII	ISI	$1.393\times 10^{-16}$
MW	MIRI	ISI	$1.766\times 10^{-16}$
MW	MRI	ISI	$2.273\times 10^{-16}$
MW	MDI	ISI	$2.191\times 10^{-16}$
MW	MHI	ISI	$1.799\times 10^{-16}$
MW	MMRI	ISI	$1.969\times 10^{-16}$
MW	MMRDI	ISI	$2.273\times 10^{-16}$
MW	MMDI	MMSDI	$8.876\times 10^{-13}$
MW	MMDI	ISI	$2.183\times 10^{-16}$
MW	MMSDI	ISI	$2.146\times 10^{-16}$
MW	SDDI	ISI	$2.267\times 10^{-16}$

Among them, the combination of MLSI and ISI yielded the lowest p -value and the highest $R^{2}$ (0.999), establishing it as the most statistically sound model for MW. This near-perfect performance indicates that the complex structural information encoded by ISI (related to connectivity and branching) is synergistically complemented by MLSI (related to the topological distance), allowing the model to almost perfectly capture molecular size. ISI and MMSDI emerged as key predictors, appearing frequently in top models and yielding high performance across combinations. In contrast, indices such as MRI, MMRDI, and SLI had diminished roles when ISI was present. The high frequency and statistical success of these top-performing topological indices directly form the empirical basis for generating the objective criterion weights used in the MCDM phase, thereby linking regression analysis to compound prioritization.

As observed, ISI appears as a common descriptor across most top models, indicating its robustness and predictive strength. To ensure fair consideration of all the descriptors, we examined the frequency with which each topological index appeared in the highest-performing regression models. ISI was the most frequently occurring descriptor (15 times), followed by MMSDI, MMRDI, RLI, and ISLI. Although ISI dominated the best models, this frequency distribution confirms that the contributions of other indices were not overlooked. Each descriptor was evaluated across all pairwise combinations, and its occurrence reflects its relative predictive strength rather than a limitation in model implementation.

These observations confirm the statistical validity of the modeling approach and justify the choice of the final models presented in Table 6. The current modeling efforts were limited to linear, multilinear, and quadratic regression; future work will explore logarithmic or other non-linear models to potentially enhance predictive performance for less-correlated properties.

### 8.6 MCDM-based compound ranking

**FIGURE 10:**
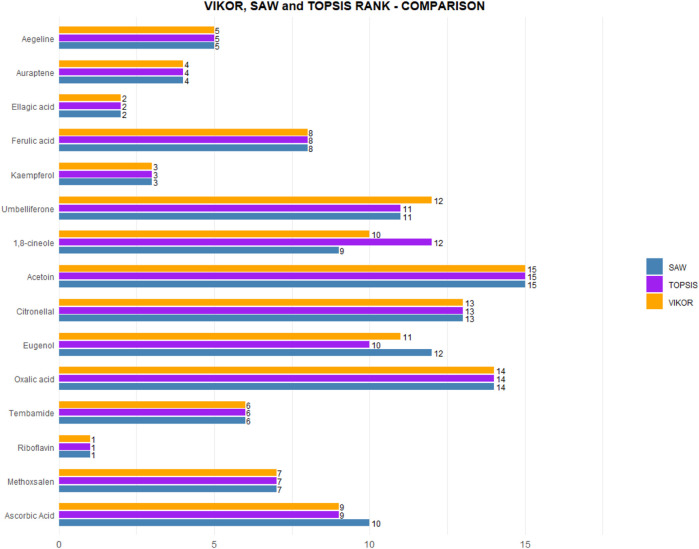
Ranking comparison of the bioactive Aegle marmelos compounds using VIKOR, SAW, and TOPSIS.

Figure 10 presents rankings of 15 bioactive compounds using three MCDM techniques—VIKOR, SAW, and TOPSIS.

High agreement was observed across all three methods. Riboflavin consistently ranked first, followed by ellagic acid and kaempferol. These compounds showed optimal balance across descriptors such as MW, TPSA, HBA/HBD, and topological indices. Lower-ranked compounds such as acetoin, citronellal, and oxalic acid consistently ranked among the bottom three, indicating poor descriptor balance.

Discrepancies (e.g., 1,8-cineole ranked ninth by VIKOR and 12th by TOPSIS) highlight method-specific differences in weighting and prioritization, but they also support the flexibility of the strategy. The final, highly concordant ranking from the three MCDM methods provides a validated, data-driven prioritization of the phytochemicals. The consistent selection of riboflavin, ellagic acid, and kaempferol as the top-three candidates is significant, as these molecules are known antioxidants and anti-inflammatory agents with established pharmacological relevance in medicinal chemistry. This computational prioritization reinforces that the QSPR-derived criteria effectively select molecules that are structurally optimized for drug-likeness, aligning the statistical model with potential therapeutic utility. This strong agreement between the initial MLR predictability results and the subsequent compound ranking from the MCDM process supports the overall robustness and reliability of the integrated framework.

The observed consistency in rankings across VIKOR, SAW, and TOPSIS indicates that the prioritization is primarily driven by intrinsic molecular characteristics rather than method-specific weighting schemes. In particular, top-ranked compounds exhibit a favorable balance between molecular size, polarity, and hydrogen-bonding functionality, while maintaining moderate structural rigidity. Conversely, lower-ranked compounds tend to lack aromatic frameworks or sufficient polar functionality, which limits their descriptor balance and reduces their suitability under QSPR-driven selection criteria. This agreement across distinct MCDM formulations reinforces the reliability of the ranking outcomes and indicates low sensitivity to minor variations in decision parameters.

### 8.7 Implications for drug development

The strong agreement across statistical models and MCDM rankings affirms the importance of TI-based regression modeling for virtual screening and QSPR tasks. Given *A. marmelos’* diverse pharmacological profile, this framework supports compound repurposing, analog synthesis, and ADMET filtering. It also facilitates translational applications in regions where bael is abundant by standardizing the descriptor-based predictions.

This approach enhances reproducibility, reduces bias via MCDM algorithms, and presents an appropriate QSPR strategy that balances simplicity (correlation) with multivariate rigor (regression). Ultimately, it supports drug-design pipelines targeting chronic conditions such as diabetes, neurological disorders, and cancers.

## 9 Conclusion

In this study, the properties of *A. marmelos* were systematically analyzed using regression-based QSPR models and multi-criteria decision-making techniques. The study focused on evaluating the predictive capacity of discrete Adriatic topological indices for modeling six key physicochemical properties, namely, molecular weight, topological polar surface area, hydrogen bond donor count, hydrogen bond acceptor count, melting point, and rotatable bond count.

Multiple linear regression models were constructed using pairwise combinations of topological indices, and their statistical quality was assessed using the standard criteria. Only models with $R^{2}\geq 0.99$ and high F-statistics were retained for further analysis. This ensured that the predictive relationships reported were statistically robust and free from multicollinearity concerns. The best-performing model was the one using MLSI and ISI to predict the MW, achieving an $R^{2}$ of 0.999 and a model p -value of $1.006\times 10^{-18}$ . Across the evaluated models, the ISI emerged as the most significant descriptor, frequently appearing in highly predictive models, especially for MW, TPSA, and HBA. This consistent predictive strength makes the ISI a critical structural criterion for the subsequent compound prioritization.

The ranking of the physicochemical property predictability, based on average $R^{2}$ values across all valid models, was established as follows:

MW>TPSA>HBA>HBD>MP>RBC.

This order emphasizes the strong structural dependencies of MW and TPSA on topological indices and the relative difficulty of modeling properties such as RBC using 2D descriptors alone.

Crucially, the major novelty of this work lies in the objective linkage between the two methodologies: the frequency with which each TI appeared in the high- $R^{2}$ QSPR models was used to derive the criterion weights for the MCDM techniques. Additionally, the study incorporated MCDM techniques such as SAW, TOPSIS, and VIKOR to prioritize 15 bioactive compounds derived from *A. marmelos*. The three ranking methods showed strong agreement, confirming the reliability of the prioritization method. Notably, riboflavin consistently ranked first across all methods, closely followed by ellagic acid and kaempferol, highlighting their superior therapeutic potential based on physicochemical properties critical for ADMET and drug-likeness. Compounds such as citronellal and oxalic acid, which scored poorly across descriptors, were consistently ranked lowest, indicating limited bioactivity alignment.

These findings underscore the efficacy of integrating statistically rigorous QSPR modeling with MCDM approaches for compound evaluation and prioritization. The methodology provides a reproducible and computationally efficient framework for high-throughput virtual screening (HTS), structural optimization, and early-phase drug discovery, offering a powerful alternative to costly experimental assays. Future work will focus on incorporating advanced non-linear machine learning models (e.g., random forests and support vector machines) and explicit 3D molecular descriptors to enhance the predictive performance for properties currently lacking robust linear models (MP and RBC).

## 10 Limitations and future directions

Despite the promising results, this study has several limitations. First, the regression models were constructed using two-variable combinations of topological indices and assumed linear relationships. Although this approach offered useful insights into descriptor performance, it may not fully capture the potential non-linear or interaction effects among the descriptors.

Second, although the topological indices were derived from MOL files that incorporated 3D molecular structures, the study did not explicitly include advanced 3D descriptors or dynamic conformational data, such as molecular fields or docking scores. These features could further enhance the predictive accuracy of QSPR models in complex biochemical systems.

Another limitation is the absence of external validation. While internal statistical reliability was demonstrated through high $R^{2}$ values and significant model p -values, validating the models on independent or experimentally verified datasets would improve their generalizability.

Additionally, the criterion weights used in the MCDM analysis were based on the frequency of occurrence of each topological index across the top-performing regression models. Although this frequency-based weighting approach is systematic and transparent, alternative methods such as entropy, CRITIC, or the analytic hierarchy process (AHP) could be explored in future studies to provide complementary insights.

For future work, the modeling framework can be expanded to include higher-order and non-linear approaches such as polynomial regression, random forests, or support vector machines, which may better capture complex descriptor–property relationships. Integration of 3D structural descriptors, conformer sampling, and pharmacologically relevant features such as docking scores or ADMET profiles would further improve biological relevance. Finally, incorporating external datasets and applying cross-validation will enhance model robustness and increase confidence in their applicability in drug development and virtual screening workflows.

## Data Availability

The raw data supporting the conclusions of this article will be made available by the authors, without undue reservation.

## References

[B1] Ahmed W. Ashraf T. Zaman S. Ullah A. Khalid F. (2025a). A hybrid computational framework for antidepressant drug design integrating machine learning algorithms and molecular modeling. Chem. Pap. 80, 589–614. 10.1007/s11696-025-04413-w

[B2] Ahmed W. Fatima G. Zaman S. Ullah A. Mahmoud E. E. Ashraf T. (2025b). Topological and statistical regression study of chemical structures using graph-theoretic descriptors: applications to cancer therapeutics. Chem. Pap. 80, 461–483. 10.1007/s11696-025-04404-x

[B3] Anuradha D. S. Jaganathan B. (2023). Physiochemical properties of benzophenone and curcumin-conjugated pamam dendrimers using topological indices. Polycycl. Aromat. Compd. 44, 3419–3441. 10.1080/10406638.2023.2234542

[B4] Anuradha D. S. Jaganathan B. (2025). Predictive modelling and ranking: azadirachta indica compounds through indices and multi-criteria decision-making techniques. Front. Chem. 13, 13–2025. 10.3389/fchem.2025.1580267 40365176 PMC12069329

[B5] Anuradha D. S. Julietraja K. Jaganathan B. Alsinai A. (2024). Curcumin-conjugated pamam dendrimers of two generations: comparative analysis of physiochemical properties using adriatic topological indices. ACS Omega 9, 14558–14579. 10.1021/acsomega.4c00686 38559925 PMC10976413

[B6] Ashraf T. Idrees N. (2024). Topological indices based vikor assisted multi-criteria decision technique for lung disorders. Front. Chem. 12, 12–2024. 10.3389/fchem.2024.1407911 39380949 PMC11459094

[B7] Balali M. Sobati M. A. Gorji A. E. (2021). Qspr modeling of thiophene distribution between deep eutectic solvent (des) and hydrocarbon phases: effect of hydrogen bond donor (hbd) structure. J. Mol. Liq. 342, 117496. 10.1016/j.molliq.2021.117496

[B8] Baliga M. S. Bhat H. P. Joseph N. Fazal F. (2011). Phytochemistry and medicinal uses of the bael fruit (Aegle marmelos correa): a concise review. Food Res. Int. 44, 1768–1775. 10.1016/j.foodres.2011.02.008

[B9] Banerjee A. Jain S. Lokesh Singh A. Dhakad A. Singh A. E. (2024). A review: medicinal properties and health benefits of bael (aegle marmelos). J. Sci. Res. Rep. 30, 773–786. 10.9734/jsrr/2024/v30i62094

[B10] Caron G. Digiesi V. Solaro S. Ermondi G. (2020). Flexibility in early drug discovery: focus on the beyond-rule-of-5 chemical space. Drug Discov. Today 25, 621–627. 10.1016/j.drudis.2020.01.012 31991117

[B11] [Dataset] ChemSpider (2025a). Chemspider: aegeline (csid: 10296259), chemical structure database.

[B12] [Dataset] ChemSpider (2025b). Chemspider: Auraptene (csid: 1267148), chemical structure database.

[B13] [Dataset] ChemSpider (2025c). Chemspider: ellagic acid (csid: 4445149), chemical structure database.

[B14] [Dataset] ChemSpider (2025d). (e)-ferulic acid | chemspider (csid: 689).

[B15] [Dataset] ChemSpider (2025e). Kaempferol | chemspider (csid: 4444395).

[B16] Choudhary S. Chaudhary G. Kaurav H. (2021). Aegle marmelos (bael patra): an ayurvedic plant with ethnomedicinal value. Int. J. Res. Ayurveda Pharm. 12, 147–156. 10.7897/2277-4343.120392

[B17] Chu K. A. Yalkowsky S. H. (2009). An interesting relationship between drug absorption and melting point. Int. J. Pharm. 373, 24–40. 10.1016/j.ijpharm.2009.01.026 19429285

[B18] Churchman C. W. Ackoff R. L. Arnoff E. L. (1957). Introduction to operations research. New York: Wiley.

[B19] Coimbra J. T. S. Feghali R. Ribeiro R. P. Ramos M. J. Fernandes P. A. (2021). The importance of intramolecular hydrogen bonds on the translocation of the small drug piracetam through a lipid bilayer. RSC Adv. 11, 899–908. 10.1039/d0ra09995c 35423709 PMC8693363

[B20] Ekor M. (2014). The growing use of herbal medicines: issues relating to adverse reactions and challenges in monitoring safety. Front. Pharmacol. 4, 177. 10.3389/fphar.2013.00177 24454289 PMC3887317

[B21] Farooq F. B. Idrees N. Noor E. Alqahtani N. A. Imran M. (2025). A computational approach to drug design for multiple sclerosis via qspr modeling, chemical graph theory, and multi-criteria decision analysis. BMC Chem. 19, 1. 10.1186/s13065-024-01374-1 39748369 PMC11697749

[B22] Harsha Vardhan K. S. Anuradha D. S. Jaganathan B. (2023). Double bond indices and their application: qsar of polycyclic aromatic hydrocarbons. Util. Math. 122, 3–27. 10.61091/um122-01

[B23] [Dataset] Human Metabolome Database (HMDB) (2022). Auraptene (HMDB0034054). Available online at: https://hmdb.ca/metabolites/HMDB0034054 (Accessed June 11, 2025).

[B24] [Dataset] Human Metabolome Database (2025a). Aegeline (hmdb0033435). Available online at: https://www.hmdb.ca/metabolites/HMDB0033435 (Accessed June 12, 2025).

[B25] [Dataset] Human Metabolome Database (2025b). Ferulic acid (hmdb0000954). Available online at: http://www.hmdb.ca/metabolites/HMDB0000954 (Accessed June 12, 2025).

[B26] Husin M. N. Khan A. R. Awan N. U. H. Campena F. J. H. Tchier F. Hussain S. (2024). Multicriteria decision making attributes and estimation of physicochemical properties of kidney cancer drugs via topological descriptors. PLOS ONE 19, 1–19. 10.1371/journal.pone.0302276 38713692 PMC11075897

[B27] Hwang C. L. Yoon K. (1981). Multiple attribute decision making: methods and applications. New York: Springer-Verlag.

[B28] Joy Prisca A. Jaganathan B. (2025). Internal functionalized drug delivery dendrimers: theoretical analysis by descriptors. Lett. Appl. NanoBioScience 14, 65.

[B29] Keyvanpour M. Shirzad M. (2020). An analysis of qsar research based on machine learning concepts. Curr. Drug Discov. Technol. 17. 10.2174/1570163817666200316104404 32178612

[B30] Khanal A. Dall’acqua S. Adhikari R. (2023). Bael (aegle marmelos), an underutilized fruit with enormous potential to be developed as a functional food product: a review. J. Food Process. Preserv. 8863630, 11. 10.1155/2023/8863630

[B31] Lionta E. Spyrou G. Vassilatis D. K. Cournia Z. (2014). Structure-based virtual screening for drug discovery: principles, applications and recent advances. Curr. Top. Med. Chem. 14, 1923–1938. 10.2174/1568026614666140929124445 25262799 PMC4443793

[B32] Macarron R. Banks M. Bojanic D. Burns D. Cirovic D. Garyantes T. (2011). Impact of high-throughput screening in biomedical research. Nat. Reviews. Drug Discovery 10, 188–195. 10.1038/nrd3368 21358738

[B33] Madanchian M. Taherdoost H. (2023). A comprehensive guide to the topsis method for multi-criteria decision making. Sustain. Soc. Dev. 1. 10.54517/ssd.v1i1.2220

[B34] Mercader A. G. Goodarzi M. Duchowicz P. R. Fernández F. M. Castro E. A. (2010). Predictive qspr study of the dissociation constants of diverse pharmaceutical compounds. Chem. Biol. and Drug Des. 76, 433–440. 10.1111/j.1747-0285.2010.01033.x 20925694

[B35] Monika S. Thirumal M. Kumar P. R. (2023). Phytochemical and biological review of Aegle marmelos Linn. (English). Future Sci. OA 1. 10.2144/fsoa-2022-0068 PMC10072075 37026028

[B36] Mujeeb F. Khan A. F. Pandey V. K. Sinha A. Barwant M. M. Rustagi S. (2025). Significance, pharmacological properties, and industrial applications of bael (aegle marmelos): a review of current knowledge. J. Agric. Food Res. 19, 101631. 10.1016/j.jafr.2025.101631

[B37] Narender T. Shweta S. Tiwari P. Papi Reddy K. Khaliq T. Prathipati P. (2007). Antihyperglycemic and antidyslipidemic agent from aegle marmelos. Bioorg. and Med. Chem. Lett. 17, 1808–1811. 10.1016/j.bmcl.2006.12.037 17197179

[B38] [Dataset] National Center for Biotechnology Information (2025a). Pubchem compound summary for cid 445858, ferulic acid. Available online at: https://pubchem.ncbi.nlm.nih.gov/compound/Ferulic-acid (Accessed June 12, 2025).

[B39] [Dataset] National Center for Biotechnology Information (2025b). Pubchem compound summary for cid 5281855, ellagic acid. Available online at: https://pubchem.ncbi.nlm.nih.gov/compound/Ellagic-Acid (Accessed June 12, 2025).

[B40] [Dataset National Toxicology Program (1992). “National toxicology program chemical repository database,” in National Institute of Environmental Health Sciences (Research Triangle Park, NC: National Institutes of Health).

[B41] Niazi S. K. Mariam Z. (2023). Recent advances in machine-learning-based chemoinformatics: a comprehensive review. Int. J. Mol. Sci. 24, 11488. 10.3390/ijms241411488 37511247 PMC10380192

[B42] Opricovic S. Tzeng G. H. (2004). Compromise solution by mcdm methods: a comparative analysis of vikor and topsis. Eur. J. Operational Res. 156, 445–455. 10.1016/j.ejor.2003.12.032

[B43] Peng X. Gibbs E. Silverman J. M. Cashman N. R. Plotkin S. S. (2021). A method for systematically ranking therapeutic drug candidates using multiple uncertain screening criteria. Stat. Methods Med. Res. 30, 1502–1522. 10.1177/09622802211002861 33847541 PMC8189013

[B44] Prasanna S. Doerksen R. J. (2009). Topological polar surface area: a useful descriptor in 2d-qsar. Curr. Med. Chem. 16, 21–41. 10.2174/092986709787002817 19149561 PMC7549127

[B45] [Dataset] PubChem (2025a). Acetoin (3-hydroxy-2-butanone). Available online at: https://pubchem.ncbi.nlm.nih.gov/compound/Acetoin#section=Boiling-Point (Accessed July 03, 2025).

[B46] [Dataset] PubChem (2025b). Aegelin. PubChem compound database.

[B47] [Dataset] PubChem (2025c). Ascorbic acid. Available online at: https://pubchem.ncbi.nlm.nih.gov/compound/Ascorbic-Acid#section=Odor (Accessed July 4, 2025).

[B48] [Dataset] PubChem (2025d). Auraptene (CID 1550607). Available online at: https://pubchem.ncbi.nlm.nih.gov/compound/1550607 (Accessed June 11, 2025).

[B49] [Dataset] PubChem (2025e). Citronellal. Available online at: https://pubchem.ncbi.nlm.nih.gov/compound/Citronellal#section=Boiling-Point (Accessed July 3, 2025).

[B50] [Dataset] PubChem (2025f). Eucalyptol. Available online at: https://pubchem.ncbi.nlm.nih.gov/compound/2758 (Accessed June 03, 2025).

[B51] [Dataset] PubChem (2025g). Eugenol. Available online at: https://pubchem.ncbi.nlm.nih.gov/compound/Eugenol#section=Boiling-Point (Accessed July 3, 2025).

[B52] [Dataset] PubChem (2025h). Kaempferol.

[B53] [Dataset] PubChem (2025i). Methoxsalen. Available online at: https://pubchem.ncbi.nlm.nih.gov/compound/Methoxsalen#section=Odor (Accessed July 4, 2025).

[B54] [Dataset] PubChem (2025j). Oxalic acid. Available online at: https://pubchem.ncbi.nlm.nih.gov/compound/Oxalic-Acid#section=Boiling-Point (Accessed July 3, 2025).

[B55] [Dataset] PubChem (2025k). Riboflavin. Available online at: https://pubchem.ncbi.nlm.nih.gov/compound/Riboflavin#section=Odor (Accessed July 3, 2025).

[B56] [Dataset] PubChem (2025l). Tembamide. Available online at: https://pubchem.ncbi.nlm.nih.gov/compound/Tembamide#section=Experimental-Properties (Accessed July 3, 2025).

[B57] [Dataset] PubChem, N. (2025). Umbelliferone (pubchem compound cid 5281426). Available online at: https://pubchem.ncbi.nlm.nih.gov/compound/Umbelliferone (Accessed on July 3, 2025).

[B58] Rahman M. T. Halim M. A. Mozumder N. R. Ove T. A. Khatun A. A. (2024). Phytochemicals and antioxidant properties of bael (aegle marmelos l.) pulp powder and its products. J. Agric. Food Res. 15, 100971. 10.1016/j.jafr.2024.100971

[B59] Randic M. (1975). Characterization of molecular branching. J. Am. Chem. Soc. 97, 6609–6615. 10.1021/ja00856a001

[B60] [Dataset] Royal Society of Chemistry (2025a). Chemspider: chemical structure database. Available online at: https://www.chemspider.com/Chemical-Structure.10189562.html (Accessed July 5, 2025).Csid 10189562

[B61] [Dataset] Royal Society of Chemistry (2025b). Chemspider: chemical structure database, csid. Available online at: https://www.chemspider.com/Chemical-Structure.13876103.html (Accessed July 5, 2025).13876103

[B62] [Dataset] Royal Society of Chemistry (2025c). Chemspider: chemical structure database, csid. Available online at: https://www.chemspider.com/Chemical-Structure.154600.html (Accessed July 5, 2025).154600

[B63] [Dataset] Royal Society of Chemistry (2025d). Chemspider: chemical structure database, csid. Available online at: https://www.chemspider.com/Chemical-Structure.21105851.html (Accessed July 5, 2025).21105851

[B64] [Dataset] Royal Society of Chemistry (2025e). Chemspider: chemical structure database, csid, 2656. Available online at: https://www.chemspider.com/Chemical-Structure.2656.html (Accessed July 5, 2025).

[B65] [Dataset] Royal Society of Chemistry (2025f). Chemspider: chemical structure database, csid, 3971. Available online at: https://www.chemspider.com/Chemical-Structure.3971.html (Accessed July 5, 2025).

[B66] [Dataset] Royal Society of Chemistry (2025g). Chemspider: chemical structure database, csid. Available online at: https://www.chemspider.com/Chemical-Structure.431981.html (Accessed July 5, 2025).431981

[B67] [Dataset] Royal Society of Chemistry (2025h). Chemspider: chemical structure database, csid. Available online at: https://www.chemspider.com/Chemical-Structure.4444774.html (Accessed July 5, 2025).4444774

[B68] [Dataset] Royal Society of Chemistry (2025i). Chemspider: chemical structure database, csid, 7506. Available online at: https://www.chemspider.com/Chemical-Structure.7506.html (Accessed July 5, 2025).

[B69] [Dataset] Royal Society of Chemistry (2025j). Chemspider: chemical structure database, csid, 946. Available online at: https://www.chemspider.com/Chemical-Structure.946.html (Accessed July 5, 2025).

[B70] Sathyanarayanan A. D. Jaganathan B. (2023). Topological properties of boron triangular sheet for robotic finger flex motion through indices. AIP Conf. Proc. 2946, 020013. 10.1063/5.0178070

[B71] Shanmukha M. C. Basavarajappa N. S. Shilpa K. C. Usha A. (2020). Degree-based topological indices on anticancer drugs with qspr analysis. Heliyon 6, e04235. 10.1016/j.heliyon.2020.e04235 32613116 PMC7322043

[B72] Shino Y. Kaneko H. (2025). Improving molecular design with direct inverse analysis of qsar/qspr model. Mol. Inf. 44, e202400227. 10.1002/minf.202400227 39797757 PMC11724648

[B73] Shirakol S. Kalyanshetti M. Hosamani S. M. (2019). Qspr analysis of certain distance based topological indices. Appl. Math. Nonlinear Sci. 4, 371–386. 10.2478/AMNS.2019.2.00032

[B74] Singh A. Singh S. Saroj P. Krishna H. Singh R. Singh R. (2019). Research status of bael (aegle marmelos) in India: a review. Indian J. Agric. Sci. 89, 1563–1571. 10.56093/ijas.v89i10.94576

[B75] Taherdoost H. (2023). Analysis of simple additive weighting method (saw) as a multi-attribute decision making technique: a step-by-step guide. J. Manag. Sci. and Eng. Res. 6, 21–24. 10.30564/jmser.v6i1.5400

[B76] Toews P. Bates J. (2023). Influence of drug and polymer molecular weight on release kinetics from hema and hpma hydrogels. Sci. Rep. 13, 16685. 10.1038/s41598-023-42923-3 37794078 PMC10550905

[B77] Vukicevic D. (2011). Bond additive modeling 4. qspr and qsar studies of the variable adriatic indices. Croat. Chem. Acta 84, 87–91. 10.5562/cca1666

[B78] Vukičević D. Gašperov M. (2010). Bond additive modeling 1. adriatic indices. Croat. Chem. Acta 83, 243–260.

[B79] Yadav M. Rana A. (2025). Aegle marmelos (l.): an underutilized plant with incredible pharmaceutical and nutritional potential. Food Chem. Adv. 6, 100935. 10.1016/j.focha.2025.100935

